# Combinatorial protein dimerization enables precise multi-input synthetic computations

**DOI:** 10.1038/s41589-023-01281-x

**Published:** 2023-03-09

**Authors:** Adrian Bertschi, Pengli Wang, Silvia Galvan, Ana Palma Teixeira, Martin Fussenegger

**Affiliations:** 1 Department of Biosystems Science and Engineering, ETH Zurich, Basel, Switzerland; 2 University of Basel, Faculty of Science, Basel, Switzerland

**Keywords:** Synthetic biology, Transcription

## Abstract

Bacterial transcription factors (TFs) with helix-turn-helix (HTH) DNA-binding domains have been widely explored to build orthogonal transcriptional regulation systems in mammalian cells. Here we capitalize on the modular structure of these proteins to build a framework for multi-input logic gates relying on serial combinations of inducible protein–protein interactions. We found that for some TFs, their HTH domain alone is sufficient for DNA binding. By fusing the HTH domain to TFs, we established dimerization dependent rather than DNA-binding-dependent activation. This enabled us to convert gene switches from OFF-type into more widely applicable ON-type systems and to create mammalian gene switches responsive to new inducers. By combining both OFF and ON modes of action, we built a compact, high-performance bandpass filter. Furthermore, we were able to show cytosolic and extracellular dimerization. Cascading up to five pairwise fusion proteins yielded robust multi-input AND logic gates. Combinations of different pairwise fusion proteins afforded a variety of 4-input 1-output AND and OR logic gate configurations.

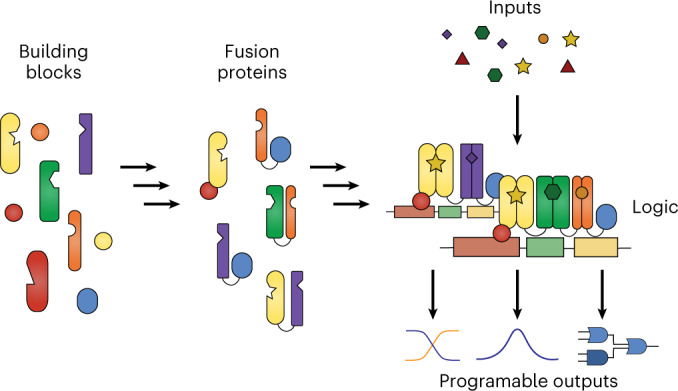

## Abstract (web summary)

The authors show modular functionality of TetR-like proteins in mammalian cells, separating the protein–DNA and the protein–protein interaction. This allows for engineered ON- and OFF-type responses to stimuli, higher order and multi-input logics.

## Main

Living cells interact with their surroundings and with each other by recognizing specific molecular gradients in complex chemical mixtures, followed by logical processing of the sensed information. This processing depends on genetically encoded programs and is often reliant on binary functions^1–3^. Synthetic design of such decision-making programs in mammalian cells could open up new applications in tissue engineering^4^, stem cell differentiation^5,6^ and gene therapy^7,8^. The electronic counterparts of such regulatory networks are combinations of a few fundamental switches, which can be employed in various sequences to obtain the desired logical downstream processing. However, while electronic circuits can be shielded by insulating layers, cells have to be able to detect specific targets in complex mixtures to achieve higher-order logical processing. Complex transcription-based logic circuits have been built in mammalian cells, based on multiple reporter regions^9–12^ or constitutive dimerization^13^, but they suffer from issues such as long induction times, low design flexibility and/or lack of inducibility. In particular, the paucity of inducible dimerization proteins that can be multiplexed restricts design flexibility.

In this study, we focused on one of the largest small-molecule-responsive protein families, the helix-turn-helix (HTH) transcription factors (TFs), as building blocks to create large digital circuits in mammalian cells. The orthogonality of many of these bacterial transcriptional regulators is well established in mammalian cells^14^. During evolution, members of this protein family have acquired the ability to specifically recognize various small molecules^15^, such as sugars^16^, amino acids^17,18^, vitamins^19^ and other metabolites^20^. They share a common structure of the DNA-binding domain (DBD), which consists of three alpha helixes connected by small linkers^21^. While the functionality of these proteins can vary greatly in bacteria, often including sigma recruiting factors or other transregulatory domains, most of these functions are absent in mammalian cells, which lack many of the interacting proteins. The two domains that are effectively used in mammalian cells are the DBD and the effector binding domain (EBD). We found that the truncated DBD alone of some TFs still binds strongly to the cognate DNA-binding site, and that whole TF can dimerize in the presence of their effectors without requiring DNA interaction. Capitalizing on these features and integrating other chemically induced heterodimerization systems, we developed large orthogonal gates based on inducer-controlled cascades of protein fusions (LOGIC). By changing the protein–protein fusions, we were able to create all combinations of AND and OR gate switches, enabling modular logic gate design of up to 4-input logic circuits with robust performance.

This framework can fundamentally change gene switches natively functioning as OFF systems, in which the presence of the effector molecule disturbs binding of the TF fused to a transcriptional activator (TA) domain to the output promoter, thereby switching gene expression OFF. Specifically, these OFF systems are converted to ON switches, as the presence of the effector allows dimerization of the TF, colocalizing the fused TA in the output promoter to activate gene expression. By integrating both ON and OFF systems responsive to the same molecule, we were able to design a bandpass filter that leads to activation only in a certain range of effector concentration, mimicking a common feature of cell differentiation^22^ and other natural regulatory mechanisms^23^.

## Results

### LOGIC design and screening of its enabling modules

**Fig. 1:**
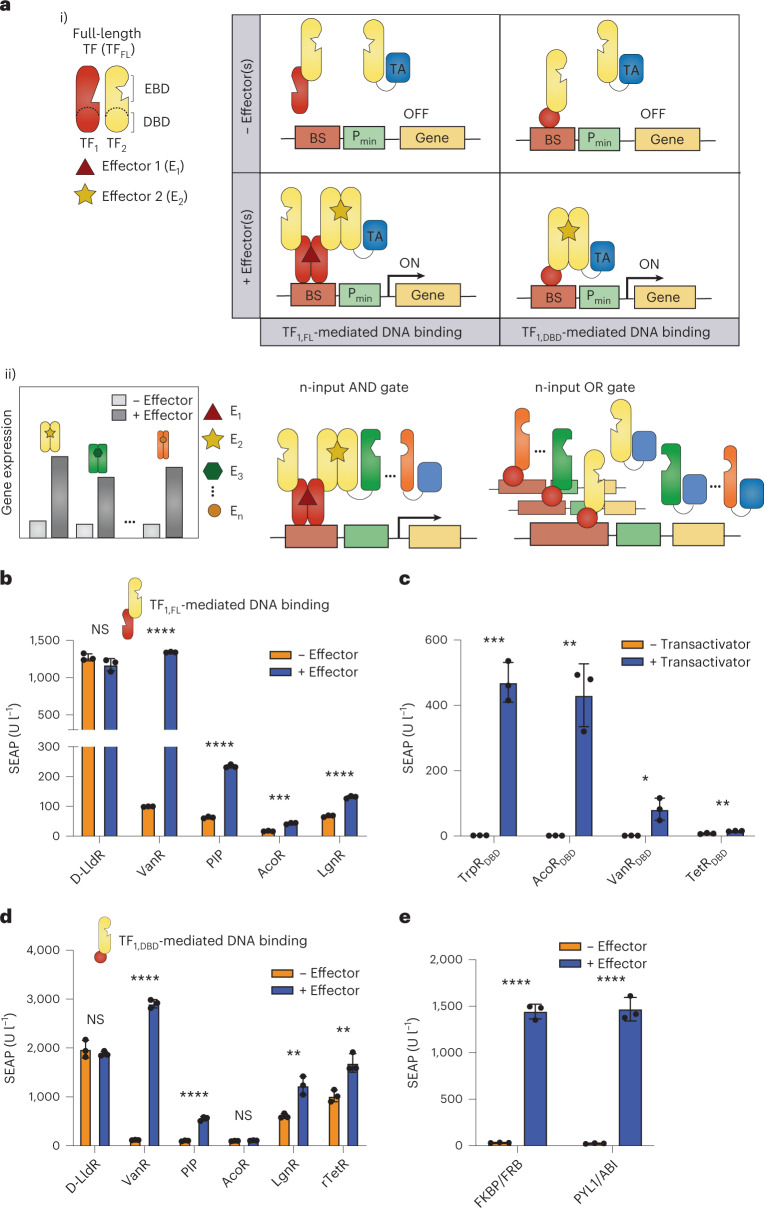
Modular design of TFs. **a**, Schematic overview of LOGIC. Modular bacterial TFs bearing an HTH DBD and an EBD are harnessed by using either a full-length TF (TF_1,FL_) or only its truncated DBD (TF_1,DBD_) to mediate specific DNA-binding and activate the output promotor (Fig. 1a, part i). This design can be used to build n-input AND logic gates, in which TF_1,FL_ or TF_1,DBD_ (in red) are fused to one or more TFs. This cascade of protein fusions ends with the nth dimerization pair (in orange), which has a partner fused to a TA domain (in blue). Multiple dimerization proteins can be fused to the same TF_1,FL_ or TF_1,DBD_ to afford n-input OR logic gates (Fig. 1a, part ii). The simultaneous formation of homodimers is omitted in the illustration for clarity. **b**, SEAP expression from HEK293T cells coexpressing two fusion proteins consisting of an HTH-containing TF (D-LldR (*P* = 0.145), VanR, PIP, AcoR (*P* = 0.0001) or LgnR) fused to either TetR or VPR. SEAP expression with and without the corresponding effector molecule was analyzed (d-lactate (25 mM), VA (250 µM), pristinamycin (10 µM), acetoin (10 mM), d-idonate (1 mM)). **c**, SEAP expression from HEK293T cells cotransfected with a truncated DBD from TrpR (*P* = 0.0002), AcoR (*P* = 0.0015), VanR (*P* = 0.0149) or TetR (*P* = 0.0031) fused to the VPR TA, and the corresponding DNA-binding sites upstream of the reporter gene. **d**, SEAP expression from HEK293T cells co-expressing two fusion proteins consisting of an HTH-containing TF (D-LldR (*P* = 0.428), VanR, PIP, AcoR (*P* = 0.104), LgnR (*P* = 0.005) or rTetR (*P* = 0.007)) fused to either TrpR_DBD_ or VPR in response to the effector molecule. The concentrations used were the same as in **b**. **e**, SEAP expression from HEK293T cells coexpressing the rapamycin- or abscisic acid-inducible dimerization domains (FKBP/FRB or ABI/PYLI) fused to either TrpR_DBD_ or VPR. SEAP expression was analyzed in the presence or absence of rapamycin (50 nM) or abscisic acid (40 µM), respectively. Columns and bars in **b**–**e** indicate the mean and SD of three biological replicates, shown as solid circles. NS; not significant. *P < 0.1; **P < 0.01; ***P < 0.001; ****P < 0.0001. Two-tailed unpaired student *t*-tests were used for statistical analysis. Source data

To enable complex synthetic logic functions in mammalian cells, we first explored the modularity of bacterial TFs consisting of two domains, an HTH DBD and an EBD, which function as dimers during activation or repression of gene expression. For some TFs, dimerization is induced by effector binding, thereby making the TF DNA-binding-competent, while for others, effector binding induces structural changes in the dimer complexes, altering the affinity for DNA. We devised two strategies to use these TFs as building blocks of synthetic circuits to regulate transgene expression in mammalian cells in different ways. We fused either a full-length TF (TF_1,FL_) or its DBD alone (TF_1,DBD_) to a second full-length TF (TF_2,FL_; Fig. 1a, part i). Then, effector (E_2_)-induced dimerization will bring a partner TF_2_ fused to a transactivation domain (TA) into the vicinity of the DNA-binding sequence (BS) of TF_1_, thereby activating transcription of the downstream gene. When full-length TF_1_ is employed, the system can be responsive to two effectors simultaneously, E_1_ and E_2_, constituting a 2-input logic gate. This framework, which we named LOGIC, can easily be expanded to engineer logic gates with many inputs (Fig. 1a, part ii). For instance, we can create an n-input AND gate by fusing in series several pairwise dimerization domains, in which the final nth inducible dimerization pair brings the TA close to the promoter region to activate gene expression only when all n-inputs are present. As we have decoupled DNA binding from the effector binding, we can flexibly switch any AND gate to an n-input OR gate by allowing dimerization at the same DNA origin.

**Extended Data Fig. 1:**
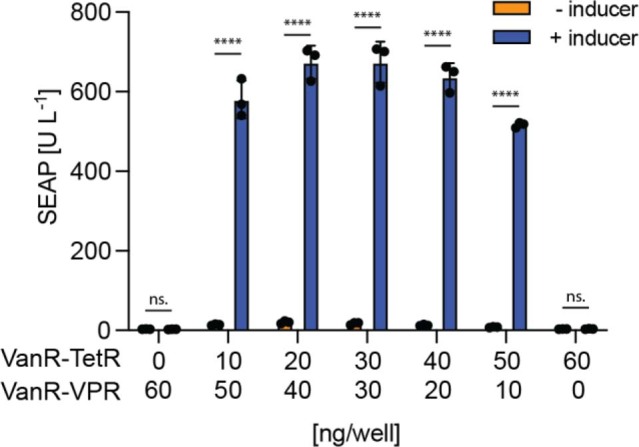
Effect of non-productive homodimers. SEAP expression from cells transfected with the VA-inducible gene switch using different ratios of the two heterodimerizing proteins VanR-TetR and VanR-VPR to assess the effect of non-productive homodimer formation on switch performance. Transfected cells were cultured in the absence or presence of VA 250 µM. Exact p-values for VanR-TetR: VanR-VPR ratios of 0:60 and 60:0 are p = 0.207 and p = 0.752 respectively. Bars indicate the mean and S.D. of three biological replicates shown as black circles. n.s., not significant; ****, *P* < 0.0001 vs the indicated condition. Two-tailed unpaired student t-tests were used for statistical analysis. Source data

We first selected the tetracycline-responsive protein TetR as full-length TF_1_, which binds to its cognate DNA BS in the absence of doxycycline (Dox). We tested five different TFs of the HTH family as TF_2_, namely the d-lactate-, vanillic acid (VA)-, pristinamycin-, acetoin- and d-idonate-responsive proteins D-LldR, VanR, PIP, AcoR^24^ and LgnR, respectively (Fig. 1b). To do so, we transiently transfected HEK293T cells with plasmids constitutively expressing the two fusion proteins (TetR-TF_2_ and TF_2_-VPR, in which VPR consists of three mammalian TAs fused together) and a TetR-responsive promoter driving the expression of the reporter protein human placental secreted alkaline phosphatase (SEAP). Cells were then cultured in the presence or absence of the corresponding effectors. While SEAP expression was fully active in the D-LldR-based system, regardless of the presence of its effector, the remaining four systems could respond to the presence of their effectors by increasing SEAP production to different extents (Fig. 1b). The VanR system, which natively functions as an OFF system, releasing VanR-VPR from its operator DNA sequence in the presence of VA, was the best performing ON switch in our LOGIC design, with 13.5-fold induction of SEAP. The LOGIC design also permits nonfunctional homodimerization of the fusion proteins, which could amount to half of the dimerized fusion proteins when using the same amount of each fusion partner. Nevertheless, the fold changes in SEAP expression in the presence of VA were only slightly impacted when we tested different ratios (between 0.2 and 5) of the two proteins VanR-TetR and VanR-VPR, indicating that nonfunctional homodimers do not substantially affect the functionality or performance of the system (Extended Data Fig. 1).

Next, we isolated the HTH DBD from four different TFs based on previously reported 3D structures on SWISS-MODEL^25–29^, including the tryptophan-responsive TrpR, AcoR, VanR and TetR, and examined whether they could still bind their cognate DNA BSs to activate gene expression when fused to the strong VPR transactivator (Fig. 1c). We found that while three of them were able to activate SEAP expression to some degree, the two best performing DBDs were those derived from TrpR (TrpR_DBD_) and AcoR (Fig. 1c). Then we probed the same full-length TFs that we had screened before fused to TetR (Fig. 1b), now fused instead to the short TrpR_DBD_, to test their ability to activate transcription in response to their effectors in this configuration. Overall, we observed similar relative performance among the different TFs in the absence and presence of the corresponding effectors for both designs (Fig. 1b,d). Nevertheless, the fold induction for the VA-based switch was better when relying on the truncated TrpR (23.1-fold induction).

**Extended Data Fig. 2:**
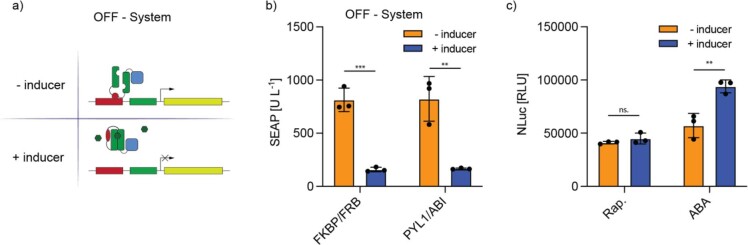
Fusion Protein based OFF system design. Fusion protein based OFF system design. **a)** Schematic illustration of the FKBP/FRB and PYL1/ABI OFF-systems. TrpR_DBD_ was flanked with either of the two heterodimerization protein pairs FKBP/FRB or PYL1/ABI, and then the mammalian transactivator VPR was added to build the fusion proteins VPR-FRB-TrpR_DBD_-FKBP or ABI-TrpR_DBD_-PYL1-VPR, respectively. In the presence of the appropriate inducer (rapamycin or abscisic acid, respectively), the proteins flanking TrpR_DBD_ dimerize, which prevents its binding to the DNA, turning gene expression OFF. **b)** SEAP expression from a TrpR-responsive promoter in HEK293T cells transfected with the FKBP/FRB (p = 0.0005) or PYL1/ABI (p = 0.0057) OFF-system, and cultured in the absence or presence of the corresponding effector molecule (rapamycin 50 µM and abscisic acid 40 µM) for 24 h. **c)** Constitutive NLuc expression in HEK293T cells co-transfected with SV40-NLuc and the FKBP/FRB (p = 0.276) or PYL1/ABI (p = 0.0078) OFF-system and cultured in the presence of rapamycin or abscisic acid, respectively, for 24 hours to examine potential toxic effects of the inducers. Bars in b and c indicate the mean and S.D. of three biological replicates, which are individually shown as solid symbols. n.s., not significant; *, *P* < 0.05; **, *P* < 0.01; ***, *P* < 0.001, vs no inducer. Two-tailed unpaired student t-tests were used for statistical analysis. Source data

Finally, we also validated this design with rapamycin- and abscisic acid (ABA)-responsive heterodimerizing domains. Fusions of each binding partner to either the TrpR_DBD_ (TrpR_DBD_-FKBP or TrpR_DBD_-ABI) or VPR (FRB-VPR or PYL1-VPR) showed strong activation of SEAP expression in the presence of each dimerization inducer (Fig. 1e). As the TrpR_DBD_ domain consists of three alpha-helix domains, we anticipated that distortion of this domain would affect its binding ability to its operator site. We therefore C-terminally fused VPR-FRB/ABI-TrpR_DBD_ to TrpR_DBD_-FRB/PYL1-VPR to obtain the four-component fusion proteins VPR-FRB-TrpR_DBD_-FKBP and ABI-TrpR_DBD_-PYL1-VPR (Extended Data Fig. 2a) and were able to demonstrate OFF switching of gene expression in the presence of rapamycin or abscisic acid, respectively (Extended Data Fig. 2b). To ensure that the OFF switching is not caused by toxicity of the inducers, we constitutively co-expressed nanoluciferase (NLuc; Extended Data Fig. 2c).

### New mammalian gene switches responsive to small molecules

**Fig. 2:**
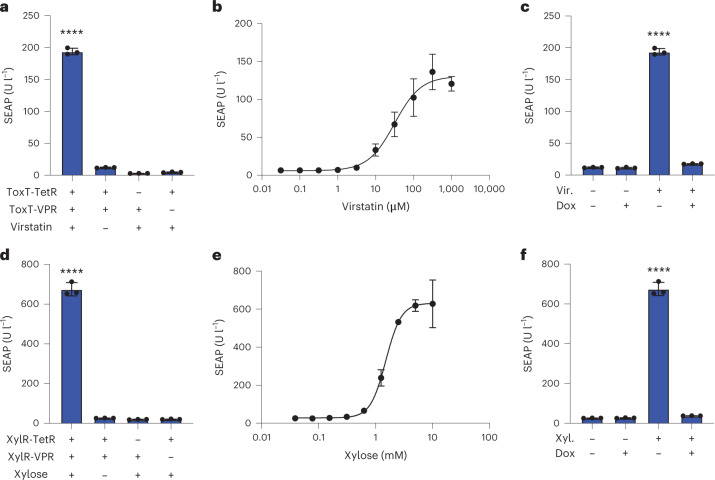
Two new mammalian gene switches responsive to virstatin and xylose. **a**, SEAP expression from HEK293T cells transfected with a TetO_7_ reporter constructs along with constitutive expression of either ToxT-TetR or ToxT-VPR, or both fusion constructs, in the presence or absence of virstatin (50 µM). **b**, Dose-dependent induction of SEAP expression in response to virstatin. HEK293T cells constitutively expressing the two proteins ToxT-TetR and ToxT-VPR, and SEAP from a TetO_7_ promoter were incubated with different concentrations of virstatin for 24 h. **c**, Two-input logic gate responsive to Dox and virstatin. HEK293T cells engineered as shown in **b** were cultured in the presence or absence of each inducer, as indicated. **d**, SEAP expression from HEK293T cells transfected with a TetO_7_ reporter construct along with constitutive expression of either XylR-TetR or XylR-VPR, or both fusion constructs, in the presence or absence of xylose (5 mM). **e**, Dose-dependent induction of SEAP expression in response to xylose. HEK293T cells constitutively expressing the two proteins XylR-TetR and XylR-VPR, and SEAP from a TetO_7_ promoter were incubated with different concentrations of xylose for 24 h. **f**, Two-input logic gate responsive to Dox and xylose. HEK293T cells engineered as shown in **d** were cultured in the presence or absence of each inducer, as indicated. Columns and bars in **a**, **c**, **d** and **f** indicate the mean and SD of three biological replicates, shown as solid circles. Data points and bars in **b** and **e** indicate the mean and SD of three biological replicates. ****P < 0.0001. Ordinary one-way ANOVA was performed for statistical analysis. Source data

**Extended Data Fig. 3:**
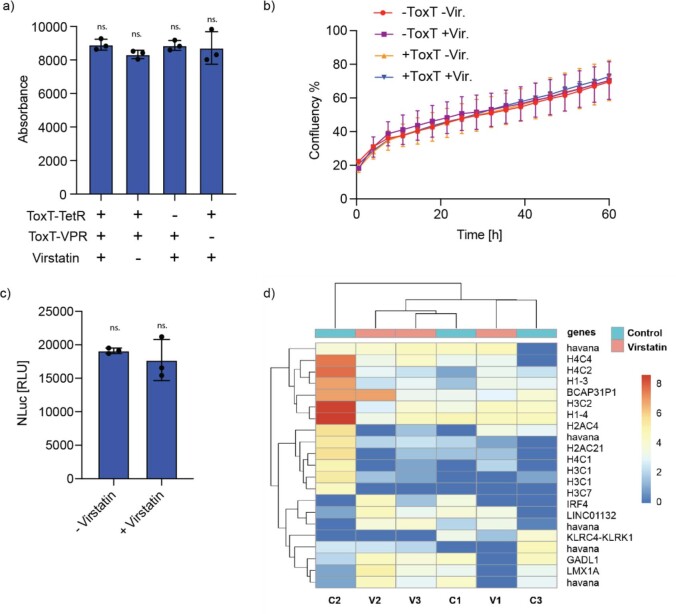
Impact of ToxT system on cellular physiology and transcriptome. Impact of the ToxT system on cellular physiology and the cellular transcriptome. **a**) Relative cell viability analysis using resazurin assay. Absorbance at 570 nm of HEK293T cell cultures expressing both parts or either part of the ToxT system and cultured in the presence or absence of virstatin, was assessed 2 hours after adding resazurin to the medium (p = 0.5817). **b**) Cell confluency analysis. HEK293T cells transfected with the ToxT system or mock plasmids and grown with or without virstatin were monitored over time by bright-field microscopy followed by image analysis to calculate the cell confluency as a measure of the cell proliferation rate. **c**) NLuc expression by cells transfected with the ToxT gene switch, along with SV40-driven constitutive expression of NLuc (P_SV40_-NLuc; pAB099), and cultured with or without virstatin (p = 0.481). **d**) RNA sequencing data of HEK293T cells transfected with the ToxT system and cultured in the presence (V1-3) or absence (C1-3) of virstatin. Hierarchical clustering with the genes with top variance of expression shows no clear separation between the two groups. Bars indicate the mean and S.D. of three biological replicates, shown as solid circles. n.s., not significant vs the indicated condition. Ordinary one-way ANOVA was performed for statistical analysis of (a) and two-tailed unpaired student t-tests was used for analysis of (c). Source data

Our framework allowed us to develop mammalian genetic switches responsive to new inducers, which are effectors of TFs from the HTH family, without the need to know the operator DNA sequence to which the TFs bind to regulate gene expression. To exemplify this, we selected two bacterial TFs that have been reported to form dimers, namely: (1) ToxT from the pathogen *Vibrio cholera*^30^ and (2) the xylose-responsive XylR from *Escherichia coli*^31,32^. ToxT is responsive to virstatin and regulates the expression of virulence factors^33,34^. We constructed fusions of ToxT to TetR and to VPR (pAB423 P_hCMV_-ToxT-TetR-pA and pAB424 P_hCMV_-ToxT-VPR-pA) and showed that transfected cells treated with virstatin increased SEAP expression by 16-fold from a tetracycline-responsive promoter (Fig. 2a). While western blot analysis of ToxT in bacterial cultures showed dimerization in the absence of virstatin^30^, our results suggest that in the context of mammalian cells, the tested ToxT fusion proteins dimerize in the presence of virstatin, allowing for activation of gene expression. The dose–response relationship of ToxT toward its inducer virstatin showed an EC 50 value of 31.5 µM (Fig. 2b), which is in the same range reported for bacteria^30^. We also showed that TetR fused to ToxT is still responsive to Dox, which can override the presence of virstatin by switching off SEAP expression (Fig. 2c). Furthermore, we confirmed that the virstatin-responsive gene switch has little impact on the cell proliferation rate, culture viability, and protein production capacity and does not substantially deregulate the mammalian transcriptome (Extended Data Fig. 3). The xylose-inducible gene switch also showed robust performance in cells constitutively expressing the two fusion proteins XylR-TetR and XylR-VPR, with SEAP expression increasing by 26-fold in the presence of 5 mM xylose (Fig. 2d). Half-maximal expression was achieved in 1.5 mM xylose-supplemented medium (Fig. 2e). As in the case of the virstatin gene switch, Dox treatment also abrogated the xylose-responsiveness of SEAP expression (Fig. 2f). Taken together, these results indicate that the LOGIC design can be used to build new small-molecule-responsive gene switches suitable for molecular computation in mammalian cells.

### Exploiting the new VA dimerization system

**Fig. 3:**
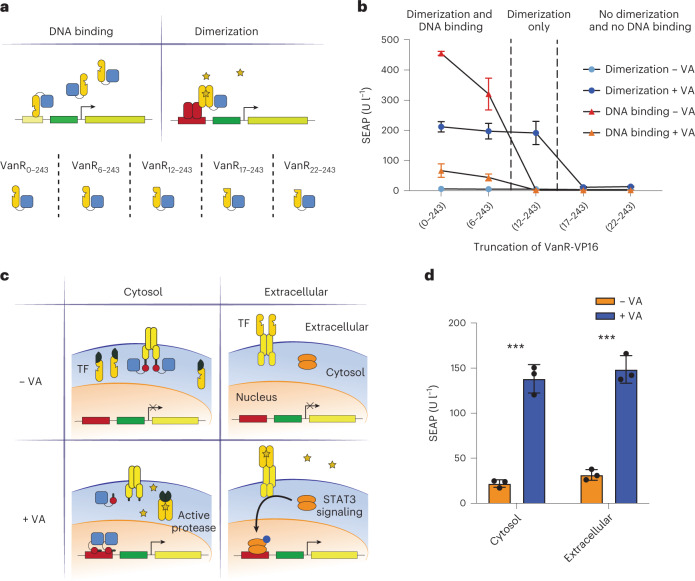
Truncation and application of VanR as a VA inducible dimer. **a**, Schematic illustration of N-terminal truncation of VanR fused to VP16 and its effect on (1) binding to its operator VanO_2_ or (2) inducing dimerization of the two fusion proteins TetR-VanR and VanR-VP16. **b**, SEAP expression from HEK293T cells transfected with the full-length VanR or four different truncated VanR variants in the presence or absence of VA. **c**, Schematic illustration of the functionality of VanR as a dimerization protein in the cytosol and extracellular space. In the cytosol, VanR-TEV_split_ dimerizes in the presence of VA, reconstituting the TEV protease that can cleave the constitutively expressed fusion membrane protein EpoR-Il6-CS_TEV_-TrpR_DBD_-VP16, releasing TrpR_DBD_-VP16, which translocates to the nucleus to activate gene expression from a TrpR-responsive promoter. On the extracellular side of the plasma membrane, the fusion protein VanR-EpoR-Il6 dimerizes in the presence of VA, which results in STAT3 activation and SEAP expression controlled by a JAK/STAT-responsive promoter. **d**, SEAP expression levels from HEK293T cells engineered with each system depicted in **c**, resulting in SEAP expression based on cytosolic (*P* = 0.0002) and extracellular (*P* = 0.0002) dimerization. Cells were incubated with or without VA for 24 h. Data points and bars in **b** indicate the mean and SD of three biological replicates. Columns and bars in **d** indicate the mean and SD of three biological replicates, shown as solid symbols. ***P < 0.001. Two-tailed unpaired student *t*-tests were used for statistical analysis. Source data

In bacteria, VanR acts as a transcriptional repressor, and binding of VA-free VanR to specific DNA operator sites is considered to block gene transcription^35^. Now, we have shown that in a mammalian cell context, the addition of VA brings VanR dimers together to activate gene transcription, relying on fusion to either the full-length TetR (Fig. 1b) or to the small TrpR DBD (Fig. 1d) and their DNA recognition sequences. Based on the 3D structure on SWISS-MODEL, the first alpha-helix of the HTH region is between Pro9 and Ser22. Therefore, to get further insight into VanR functionality, we built various truncation mutants of the VanR N-terminus, deleting the first 5, 11, 16 or 21 amino acid residues (Fig. 3a), and studied how the resulting VanR variants affect both the VA-induced dimerization system (ON system) and the VA-controlled affinity for VanO DNA sequences (OFF system; Fig. 3b). We found that the dimerization system functions equally well when using full-length VanR or VanR without the first 11 residues, but the variants without the first 16 or 21 residues (which are still part of the first alpha-helix of the HTH domain) no longer dimerize in response to VA (Fig. 3b). In contrast, the DNA-binding ability of VanR is already substantially decreased when the first five residues are removed and is completely abolished for variants without the initial 11 residues and beyond (Fig. 3b).

Next, we investigated whether the inducible VanR dimerization system also functions in other cellular contexts beyond the nucleus, namely in the cytosol and extracellular part of the plasma membrane. To do this, we fused VanR to each part of a split tobacco etch virus (TEV) protease and we fused the TrpR_DBD_-VP16 transactivator to the cytosolic part of the generalized extracellular molecule sensor platform (GEMS) receptor^36^ separated by a TEV cleavage site (pAB426; P_hCMV_-EpoR-IL6-CS_TEV_-TrpR_DBD_-VP16-pA) to provide cytosolic functionality (Fig. 3c). HEK293T cells transfected with these constructs along with the TrpR-responsive SEAP reporter showed significantly higher SEAP levels when cultured in the presence of VA (Fig. 3d). To test dimerization on the extracellular side of the plasma membrane, VanR was fused to the C-terminus of EpoR in the GEMS receptor (pAB427; P_hCMV_-VanR-EpoR-IL6-pA), which, upon dimerization, activates the STAT3 (signal transducer and activator of transcription 3) pathway and culminates in reporter gene expression driven by a synthetic promoter containing a STAT3-responsive element (Fig. 3c). SEAP production was significantly increased in transiently transfected HEK293T cells treated with VA. These results show that VA can induce dimerization of VanR in the following three cellular compartments: (1) when fused extracellularly to a synthetic receptor, (2) when it is present in the cytoplasm and (3) in the nucleus.

### Higher-order transcriptional logic gates

**Fig. 4:**
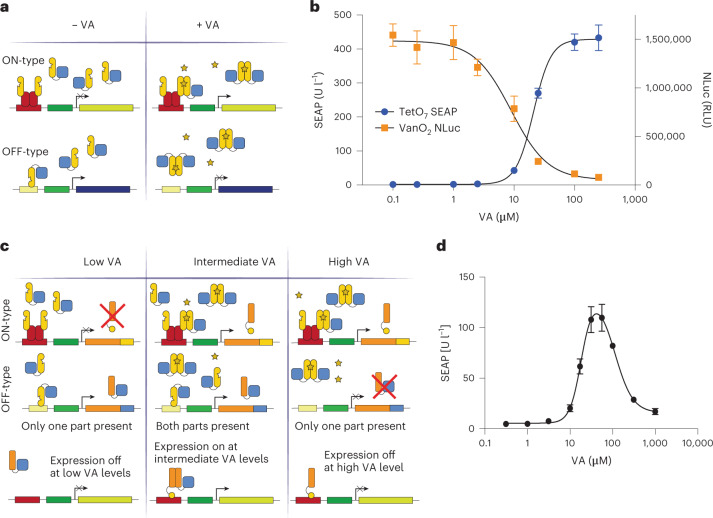
Higher-order signal processing logic circuits based on VA. **a**, Schematic illustration of a circuit converting a single input (VA) into multiple distinct outputs. In the absence of VA, VanR-VPR can bind to the VanO_2_ operator sequence and activate NLuc expression. In the presence of VA, VanR-VPR loses affinity for VanO_2_ and NLuc expression is turned OFF. At the same time, TetR-VanR and VanR-VPR dimerize in the presence of VA and activate SEAP expression downstream of a TetO_7_ promoter. **b**, VA dose-dependent expression of SEAP and NLuc. HEK293T cells were transfected with both VanR-based switch OFF and switch ON systems, consisting of four constructs (VanO_2_-P_min_-NLuc, TetO_7_-P_hCMVmin_-SEAP, P_hCMV_-TetR-VanR-pA and P_hCMV_-VanR-VPR-pA), and both reporter outputs were assayed with varying VA concentrations. **c**, Schematic illustration of a band-pass filter combining the VanR-based ON and OFF switches. The two reporter genes NLuc and SEAP in the previous configuration are replaced by the dimerization partners TrpR_DBD_-D-LldR and D-LldR-VPR, which activate reporter gene expression from a TrpR-responsive promoter. With the extra layer of transcriptional control, the reporter gene is only active when both upstream switches are active, which only holds true at intermediate VA concentrations, resulting in a bandpass filter. **d**, SEAP expression of the VA controllable bandpass filter. Data points and bars in **b** and **d** indicate the mean and SD of three biological replicates. Two-tailed unpaired student *t*-tests were used for statistical analysis. Source data

To further show the versatility of VanR-based systems, we first built a higher-order signal processing circuit to convert a single input (VA) into multiple distinct outputs. HEK293T cells transfected with both VA-inducible ON and OFF switches (Fig. 4a), in which the output signals consisted of SEAP and NLuc, respectively, showed distinct gene expression states as follows: (1) at low VA, SEAP was OFF and NLuc was ON, (2) at high VA, SEAP was ON and NLuc was OFF and (3) at intermediate VA, SEAP levels increased and NLuc levels decreased with increasing VA concentration (Fig. 4b). Thus, by replacing SEAP and NLuc outputs with a pair of dimerizing partners, we could create a band-pass filter that allows expression of the output gene only at intermediate concentrations of VA. To implement this, we harnessed the D-LldR TF, which showed ligand-independent dimerization (Fig. 1b), and replaced the output of the ON switch (SEAP) with D-LldR-VPR and the output of the OFF switch (NLuc) with TrpR_DBD_-D-LldR. When both fusion proteins are produced, they colocalize at the TrpR-responsive promoter, activating gene expression (Fig. 4c). The additional logic layer resulted in the expected bandpass behavior, with low basal expression for low and high levels of VA, and turning ON at intermediate levels of VA (Fig. 4d), where the SEAP output was over 8-fold higher than the average of the OFF states. This is arguably the simplest and best performing synthetic band-pass filter yet implemented in mammalian cells because earlier versions require the use of more complex networks of TFs and provide markedly lower performance than our current device^5,37^. Band-pass filters are commonly involved in responses to gradients of diffusible morphogens during development, inducing different cell fates at different concentrations^22^. Thus, the characteristics of our band-pass system should enable more precise gene expression patterning for tissue engineering applications.

### Multiplexing inducible protein–protein interactions

**Fig. 5:**
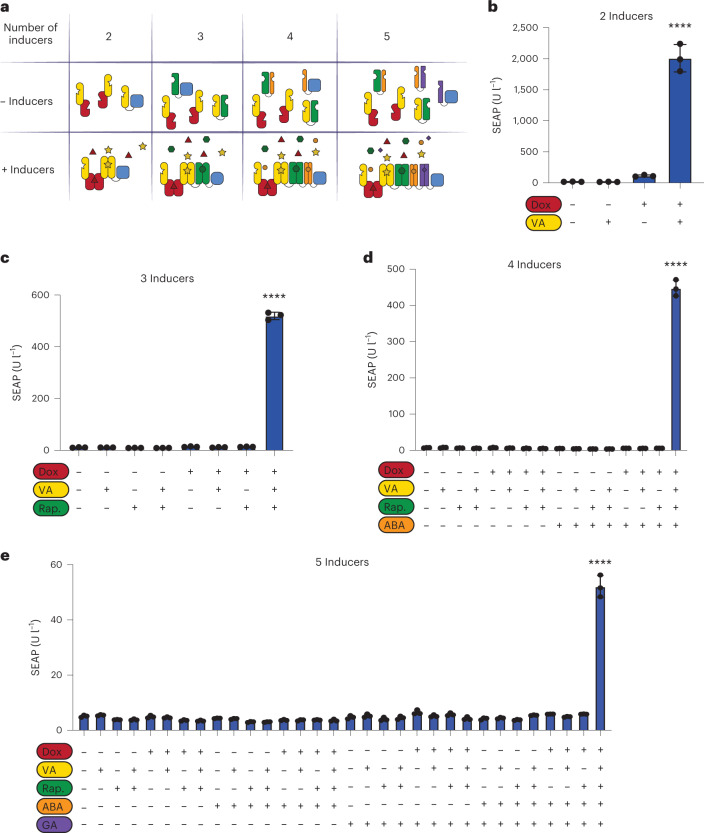
Multi-input AND logic gates based on serially arranged pairwise fusion proteins. **a**, Schematic illustration of multipartite switches responsive to 2, 3, 4 and 5 inducers. **b**, SEAP expression from a 2-input logic gate. HEK293T cells constitutively coexpressing VanR-rTetR and VanR-VPR were assayed for inducible SEAP expression in the presence or absence of Dox and VA, as indicated. **c**, SEAP expression from a 3-input logic gate. We coexpressed VanR-rTetR, FKBP-VanR and FRB-VPR and assayed inducible SEAP expression in the presence or absence of Dox, VA and Rap, as indicated. **d**, SEAP expression from a 4-input logic gate. HEK293T cells constitutively coexpressing VanR-rTetR, FKBP-VanR, FRB-PYL1 and ABI-VPR were assayed for inducible SEAP expression in the presence or absence of Dox, VA, Rap and ABA, as indicated. **e**, SEAP expression from a 5-input logic gate. We coexpressed VanR-rTetR, FKBP-VanR, FRB-ABI, PYL1-GAI and GID-VPR and assayed inducible SEAP expression in the presence or absence of Dox (red, 1 µM), VA (yellow, 250 µM), Rap (green, 50 µM), ABA (orange, 40 µM) and GA (violet, 40 µM), as indicated. Columns and bars in **b**–**e** indicate the mean and SD of three biological replicates, shown as solid circles. ****P < 0.0001. The indicated significance of the difference between the induced state and the uninduced state represents the highest *P* value obtained. Ordinary one-way ANOVA was performed for statistical analysis. Source data

**Extended Data Fig. 4:**
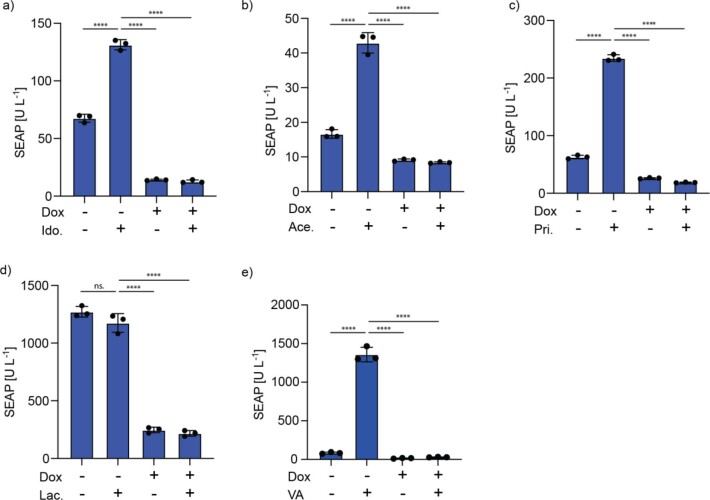
2-input Boolean logic gates. 2-input Boolean logic gates based on protein dimerization in combination with TetR. HEK293T cells were co-transfected with the TetR-responsive SEAP expression system along with constitutive expression of **a)** LgnR-TetR and LgnR-TetR, **b)** AcoR-TetR and AcoR-VPR, **c)** PIP-TetR and PIP-VPR, **d)** D-LldR-TetR and D-LldR-VPR or **e)** VanR-TetR and VanR-VPR. Transfected cells were cultured in the presence or absence of D-idonate 1 mM, acetoin 10 mM, pristinamycin 7.5 µM, D-lactate 25 mM or VA 250 µM, respectively, with or without doxycycline 1 µM, resulting in 4 different input conditions, as indicated. SEAP expression was analyzed 24 hours later to monitor the behavior of the Boolean logic gates. The exact p-value of (d) between the samples without doxycycline and with or without D-lactate is p = 0.103. Bars indicate the mean and S.D. of three biological replicates, shown as solid circles. n.s., not significant; ****, *P* < 0.0001 vs the indicated condition. Ordinary one-way ANOVA was performed for statistical analysis. Source data

**Extended Data Fig. 5:**
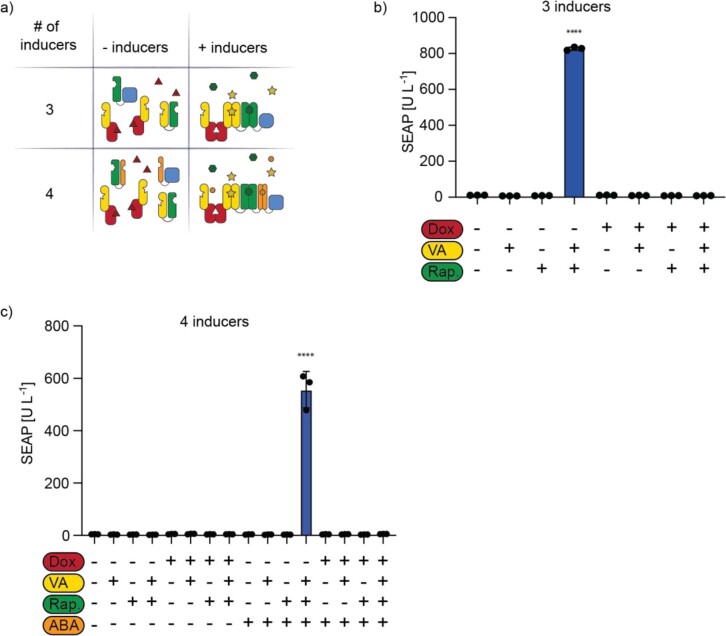
3-input and 4-input Boolean logic gates. 3-input and 4-input Boolean logic gates. **a)** Schematic illustration of the 3-input NOT A AND B AND C logic gate and 4-input NOT A AND B AND C AND D logic gates. 3- and 4-input logic gates based on protein dimerization of the three/four fusion proteins **b)** VanR-TetR, FKBP-VanR and FRB-VPR for the 3-input gate and **c)** VanR-TetR, FKBP-VanR, FRB-ABI and PYL1-VPR for the 4-input gate. Inducible SEAP expression was assayed 24 hours after induction. Cells were induced with all combinations of the presence or absence of the inducers Dox 1 µM, VA 250 µM and rapamycin 50 nM for the 3-input gate and Dox 1 µM, VA 250 µM, rapamycin 50 nM and ABA 40 µM for the 4-input gate to observe the effect of all eight/sixteen possible inducer combinations. Bars indicate the mean and S.D. of three biological replicates, shown as solid circles. ****, *P* < 0.0001 vs other conditions. Ordinary one-way ANOVA was performed for statistical analysis. Source data

As we have shown for virstatin-responsive ToxT (Fig. 2c), the fusion of TetR to another TF that dimerizes in the presence of its ligand allows us to build 2-input Boolean logic gates that can compute the presence or absence of Dox (A) and the ligand (B) into different outputs, corresponding to a NIMPLY (B AND NOT A) signal processing gate. We next characterized the performance of five additional NIMPLY gates, based on LgnR (Extended Data Fig. 4a), AcoR (Extended Data Fig. 4b), PIP (Extended Data Fig. 4c), D-LldR (Extended Data Fig. 4d) or VanR (Extended Data Fig. 4e) fused to TetR. All configurations exhibited the expected behavior for the various input combinations, considering the patterns obtained in Fig. 1b. The VanR-TetR-based gate outperformed all the others, with the ON state showing 24-fold induction in relation to the average of the OFF states. By replacing TetR with the reversed tetracycline transactivator (rTetR), we obtain 2-input AND gates, in which the signal is ON only in the presence of both inputs (Fig. 5a). The VanR-rTetR-based gate showed high performance, with the SEAP output in the presence of both inducers 42-fold above the average output of the OFF states (Fig. 5b). Therefore, we capitalized on this to expand from 2-input AND gates to 3-input, 4-input and 5-input AND gates by daisy-chaining additional protein–protein interaction domains (Fig. 5a). For the 3- and 4-input AND gates, we incorporated rapamycin-based and ABA-based heterodimerizing domains, and the resulting gates, based on constitutive expression of the three (VanR-rTetR, FKBP-VanR and FRB-VPR) or four (VanR-rTetR, FKBP-VanR, FRB-ABI and PYL1-VPR) fusions proteins, showed the expected behavior (Fig. 5c,d). Moreover, the 3-input gate B AND C AND NOT A and the 4-input gate B AND C AND D AND NOT A could be built by replacing rTetR with TetR in the previous 3-input and 4-input AND gates, resulting in average ON/OFF ratios of 47 and 83, respectively (Extended Data Fig. 5). Finally, we built a 5-input AND gate by replacing the PYL1-VPR fusion in the 4-input AND gate with PYL1-GAI and GID-VPR to incorporate gibberellic acid (GA)-dependent reporter expression. The presence of five small molecules was required to colocalize rTetR and VPR in the promoter region to activate expression of SEAP (Fig. 5e). Interestingly, when we compare the outputs of the 2-, 3- and 4-input AND gates, we can see that as the number of components increases, the OFF states become less leaky and the difference in relation to the ON states becomes greater. While the leakiness remains small for the 5-input gate, the induction is weaker compared to the lower-order input gates, resulting in an 8-fold change between the induced and the highest uninduced condition (Fig. 5e). This might be related to less stable TF complexes as the number of fusion partners increase^9,38^.

**Extended Data Fig. 6:**
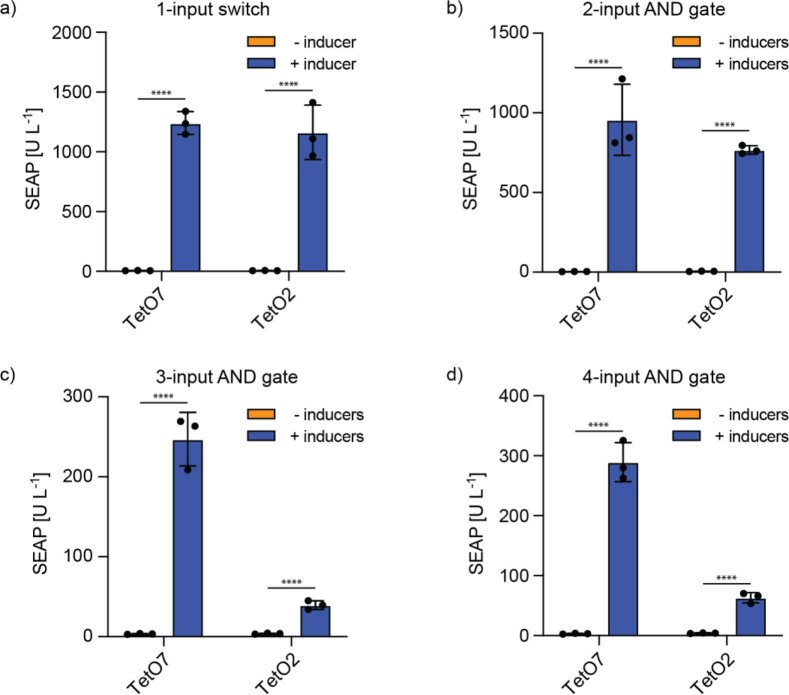
Influence of binding site on higher order logic gate performance. Performance of 1- to 4-input gene switches when using two or seven rTetR binding repeats in the promoter region of the SEAP reporter construct. **a–d** HEK293T cells were transfected with TetO7-SEAP (pVH254) or TetO2-SEAP (pAB040) combined with a) rTetR-VPR for the 1-input switch, b) VanR-rTetR and VanR-VPR for the 2-input AND gate, c) VanR-rTetR, FKBP-VanR and FRB-VPR for the 3-input AND gate, and d) VanR-rTetR, FKBP-VanR, FRB-ABI and PYL1-VPR for the 4-input AND gate. SEAP expression was analyzed after incubation for 24 h in the absence or presence of the inducers. The four effectors were used at the following concentrations: Dox 1 µM, VA 250 µM, rapamycin 50 nM, and ABA 40 µM. Bars indicate the mean and S.D. of three biological replicates, shown as solid circles. ****, *P* < 0.0001 vs the indicated condition. Two-tailed unpaired student t-tests were used for statistical analysis. Source data

Finally, to evaluate any potential correlation between the number of operator sites in the DNA-binding region and the size of the AND gates, we compared the performance of 1- to 4-input AND gates when using 2 or 7 rTetR-binding repeats (TetO_2_ and TetO_7_). While the 1- and 2-input switches had similar performances on both promoter variants, the 3- and 4-input AND gates showed higher SEAP expression levels when using the longer TetO_7_ (Extended Data Fig. 6). As the fusion protein complex is larger for higher-order input gates with more proteins involved in the complex formation, a larger DNA-binding region should provide more flexibility in DNA recognition and therefore should enable a higher number of pairwise fusion proteins to be cascaded (Extended Data Fig. 6). This suggests a potential benefit of larger promoter regions with more DNA-binding sites for higher-order AND gates.

### Four-input AND/OR logic gate combinations

**Fig. 6:**
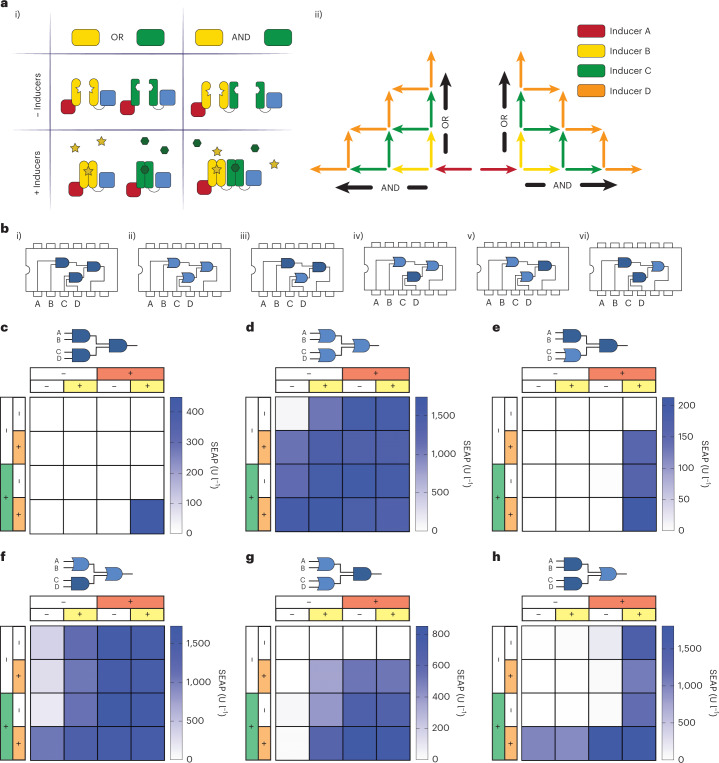
Four-input logic gates combining AND and OR operations. **a**, Schematic illustration of the fusion possibilities of multipartite switches to form logic gate combinations with up to three AND or OR gates using four small molecules as inputs to control protein–protein interactions. **b**, Schematic illustration of electronic CMOS chips for 4-input AND gate (i), 4-input OR gate (ii) and all combinations of two-level logic AND-OR designs (iii–vi). The output of a logic gate is high if both inputs are true (AND gate) or if either of the two inputs is true (OR gate). All expected outputs in regard to the four inputs are displayed in the truth table in Supplementary Table 1. **c**–**h**, Heatmap of SEAP expression from different logic gate combinations corresponding to the electronic equivalent of Fig. 6b, parts i–vi. The four inducers used were A (Dox, 1 µM), B (VA, 250 µM), C (rapamycin, 50 nM) and D (ABA, 40 µM). HEK293T cells were transfected with TetO_7_-P_min_-SEAP along with constitutive expression of different fusion proteins to achieve the desired logic, as described for each panel. **c**, (A AND B) AND (C AND D). This heatmap corresponds to the bar graph shown in Fig. 5d and is based on the four fusion components VanR-TetR, FKBP-VanR, FRB-ABI and PYL1-VPR. **d**, (A OR B) OR (C OR D) logic gate. This heatmap is based on the seven fusion proteins VanR-TetR, TetR-ABI, rTetR-VPR, VanR-VPR, FRB-VPR and PYL1-VPR. **e**, (A AND B) AND (C or D) logic gate. This heatmap is based on the five fusion proteins VanR-rTetR, VanR-ABI, FKBP-VanR, PYL1-VPR and FRB-VPR. **f**, (A OR B) OR (C AND D) logic gate. This heatmap is based on the 6 fusion proteins rTetR-VPR, VanR-TetR, VanR-VPR, TetR-FKBP, FRB-ABI and PYL1-VPR. **g**, (A OR B) AND (C OR D) logic gate. This heatmap is based on the seven fusion proteins VanR-TetR, FKBP-VanR, rTetR-FKBP, rTetR-ABI, FRB-VPR and PYL1-VPR. **h**, (A AND B) OR (C AND D) logic gate. This heatmap is based on the six fusion proteins VanR-rTetR, VanR-VPR, TetR-FKBP, rTetR-FKBP, FRB-ABI and PYL1-VPR. Bar graph diagrams of all heatmaps with statistical analysis can be found in Extended Data Fig. 8. Source data

**Extended Data Fig. 7:**
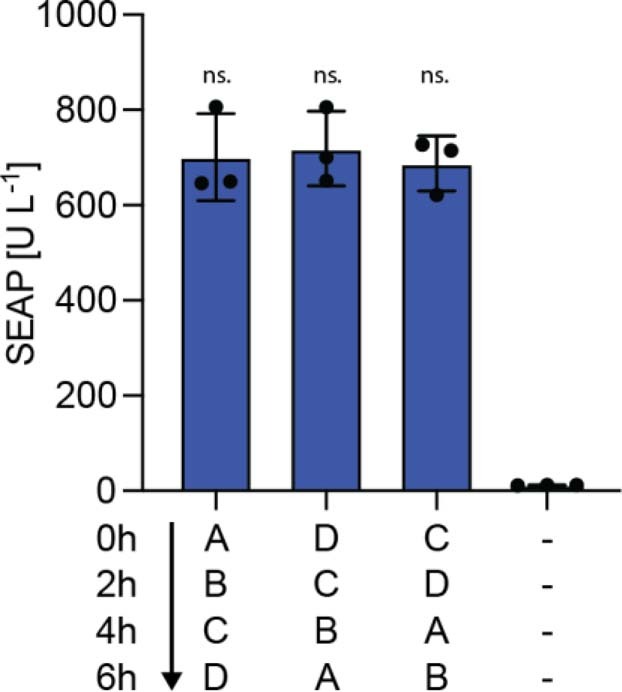
Sequential activation of muli-input gates. Performance of the 4-input AND gate upon sequential addition of the inputs at 2-hour intervals. The four inducers used were A (Dox, 1 µM), B (VA, 250 µM), C (rapamycin, 50 nM), and D (ABA, 40 µM). Ordinary one-way ANOVA of the three induced conditions results in a p-value of p = 0.759. Bars indicate the mean and S.D. of three biological replicates, shown as solid circles. n.s., not significant vs the indicated condition. Source data

**Extended Data Fig. 8:**
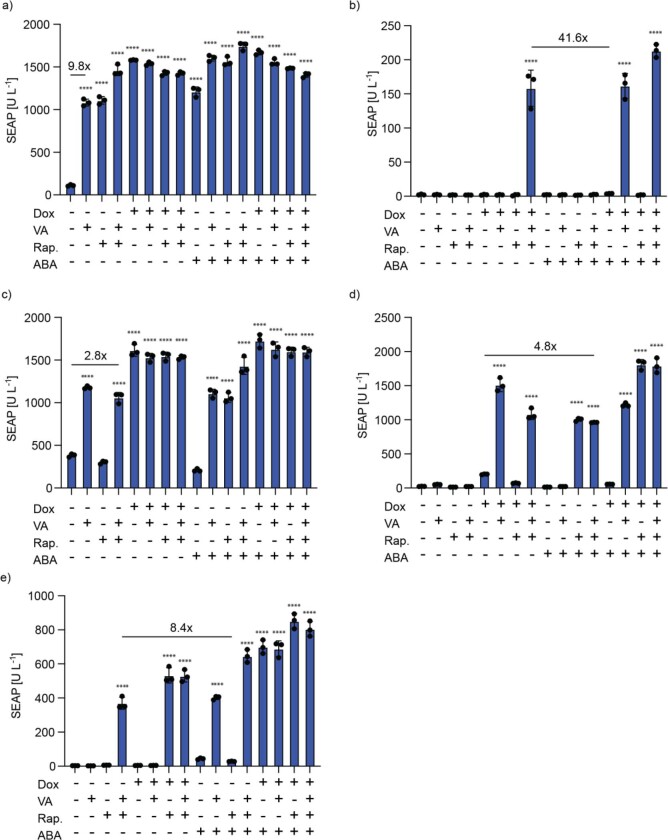
Bar-graph diagram of Logic gates. Plots a-e correspond to heatmaps of Fig. 6c–g. SEAP production in response to four small molecules controlling the protein-protein interaction of fusion proteins in different logic AND and OR gate combinations controlling a single output. Fold changes between the highest OFF state and the lowest ON state are indicated. **a)** (A OR B) OR (C OR D) logic gate. **b)** (A AND B) AND (C or D) logic gate. **c)** (A OR B) OR (C AND D) logic gate. **d)** (A OR B) AND (C OR D) logic gate. **e)** (A AND B) OR (C AND D) logic gate. The four inducers used were A (doxycycline, 1 µM), B (VA, 250 µM), C (rapamycin, 50 nM), and D (ABA, 40 µM). Bars indicate the mean and S.D. of three biological replicates, shown as solid circles. ****, *P* < 0.0001 vs no inducers. Two-tailed unpaired student t-tests were used for statistical analysis. Source data

**Extended Data Fig. 9:**
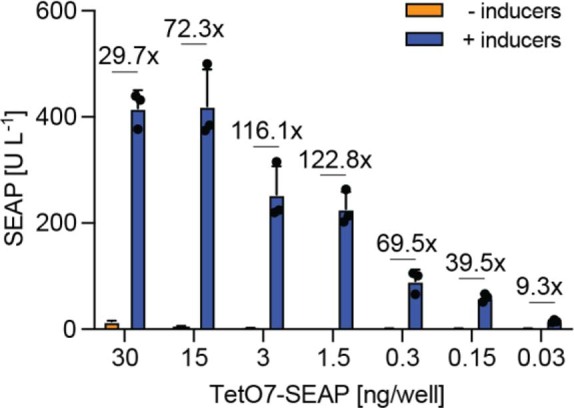
Titration of reporter plasmid of 4 input OR gate. Performance of the 4-input OR gate in cells transfected with different amounts of the reporter plasmid TetO_7_-SEAP. The concentrations of inducers used for the 4-input OR gate were Dox 1 µM, VA 250 µM, rapamycin 50 nM, and ABA 40 µM. Bars indicate the mean and S.D. of three biological replicates shown as black circles. Source data

**Extended Data Fig. 10:**
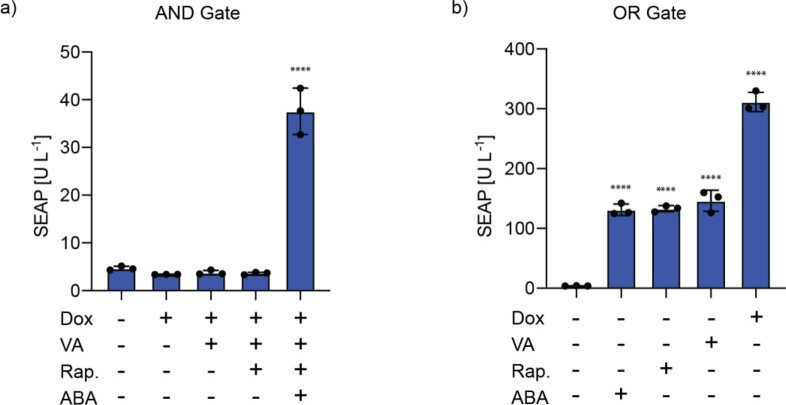
Functionality of 4-input gated on stably integrated reporter. Functionality of the 4-input **a)** AND logic and **b)** OR logic gates when the reporter construct TetO7-SEAP is stably integrated into the genome of HEK293T cells. **a)** SEAP expression from stable HEK293T-TetO_7_-SEAP cells transiently transfected with VanR-rTetR, FKBP-VanR, FRB-ABI, PYL1-VPR and cultured in the absence/presence of the indicated effector molecules. **b)** SEAP expression from stable HEK293T-TetO_7_-SEAP cells transiently transfected with rTetR-VPR, VanR-TetR, VanR-VPR, TetR-FKBP, FRB-VPR, TetR-ABI, PYL1-VPR and cultured in the absence/presence of the indicated effector molecules. The four effectors were used at the following concentrations: Dox 1 µM, VA 250 µM, rapamycin 50 nM, and ABA 40 µM. Bars indicate the mean and S.D. of three biological replicates, shown as solid circles. ****, *P* < 0.0001 vs no inducer. Two-tailed unpaired student t-tests were used for statistical analysis. Source data

By employing combinatorial arrangements of the protein components used to establish the above tetrapartite logic gates, we can create several different combinatorial 4-input 1-output AND and OR gates with a total of three gates controlling gene expression at one promoter (Fig. 6a, part i). As illustrated above, AND gates are designed by placing the pairwise dimerization domains in series, such that all pairs have to dimerize to activate gene expression. In this manner, we can freely choose a design path to a desired logic circuit by adding an AND or OR gate for each inducer (Fig. 6a, part ii). To illustrate the flexibility of this combinatorial design, the CMOS chip electronic counterparts of the 4-input AND (4082B dual 4-input AND gate) and 4-input OR gate (4072B dual 4-input OR gate), as well as multiple two-level logic circuits, are shown in Fig. 6b, parts i–vi. The respective expected outputs are displayed in the truth table. The high performance of the engineered 4-input AND gate (Fig. 5d) can also be appreciated through the SEAP expression heatmap (Fig. 6b). These results were achieved by adding the four inputs at the same time. Likewise, sequential addition of each input at 2-h intervals employing three different input sequences produced similar SEAP outputs, indicating that new dimers can be formed independently of pre-existing dimerized elements, thereby allowing the final complex to be assembled at the desired promoter region (Extended Data Fig. 7). To build OR gates, the pairwise dimerization domains are fused such that all OR-gated proteins can dimerize to the DNA-binding protein, resulting in a scenario where only one of the dimerization events is needed to colocalize the TA in the promoter region to activate gene expression (Fig. 6a). By using the ability of TetR and rTetR to bind to the same promoter region, we can flexibly design multipartite AND or OR gate switches. For instance, a four-input OR gate was designed by expressing rTetR-VPR plus six additional fusion proteins consisting of each of the three inducible dimerization pairs fused to either TetR or VPR (Fig. 6c). We found that this circuit functioned robustly, showing an average of 13-fold induction between the ON states and the OFF state (Extended Data Fig. 8a). To assess any potential effect of the reporter construct copy number on the functionality of this 4-input OR gate, we probed SEAP expression in cells transfected with decreasing doses of TetO_7_-SEAP (up to a dilution factor of 1000), keeping constant the total amount of all fusion proteins. Even using 1000-fold less reporter, we could still obtain a 9.3-fold increase of SEAP expression in the presence of the four inducers (Extended Data Fig. 9). Moreover, using a stably transgenic cell line for the TetO_7_-SEAP construct, we confirmed the functionality of the 4-input AND and OR gates in a genomic context (Extended Data Fig. 10).

Lastly, we built four additional circuits combining different AND and OR computations (Fig. 6d–g and Extended Data Fig. 8b–e), which showed the expected outcomes for the 16 inducer conditions tested. Although some gates showed some variation in SEAP expression depending on the combination of inputs, SEAP expression for the ON states was overall strong and the leakage signal of the OFF states was low.

## Discussion

Synthetically designed gene circuits are powerful tools for tissue engineering^4^, stem cell differentiation^5,6^ and gene- and cell-based therapies^7,8^. Increasing complexity achieved with modular designs is needed for application-specific processing of multiple inputs in single cells. For tissue engineering, this could lead to differentiation toward different cell types, dependent on the encoded synthetic logic^4,6^. Therefore, the development of new gene switches with different behaviors, including simple ON and OFF switches^15,39,40^, receptor platforms^36^, bandpass filters^5,37^ and logic combinations such as multi-input switches^41^ and half- and full-adders^10,42^ is attracting great interest in the field of synthetic biology.

Here we have developed a new strategy to incorporate complex synthetic computing capability into mammalian cells by exploiting bacterial TFs and other conditional dimerization systems that rely on transcriptional activation triggered by small-molecule dimerization inducers. The inducers bring the DNA-binding and transcription activation modules together, and high ON/OFF ratios were obtained with several combinations of AND and OR logic gates. While we demonstrated functionality with up to five inputs, we also identified additional HTH-containing TFs that dimerize in the presence of their effectors and therefore could be integrated to achieve even larger Boolean logic gates.

Previous attempts at molecular computation using bacterial TFs were based on transcriptional regulation of intermediate steps, resulting in long activation times and low fold changes^37^. Due to the serial design of transcriptional regulators, there is a time delay in the expression of the reporter gene. In addition, the maximum expression is limited by the inducibility of the transcriptional regulators upstream in the cascade. In contrast, multiplexing of these proteins at the expression level and regulating gene expression based on protein–protein interaction enables larger network designs in which gene expression remains high while leakiness is kept at a minimum.

To achieve multiplexing, orthogonality between different small-molecule recognition domains is a key factor. The use of a bacterial protein family as the foundation of the minimal building blocks for synthetic circuit designs allows for less intracellular crosstalk within the mammalian cells and paves the way for building multiple input switches.

Although there is a large variety of DBDs, including TALENs^43^, zinc fingers^44^ or systems based on CRISPR/dCas9 (ref. ^45^) that offer design flexibility, the truncated HTH DBDs offer a physically small alternative with strong DNA-binding properties. Due to the small structure and the specific spatial alignment of the three alpha helixes, the dimerization of two heterodimerization proteins fused at either end of the DBD can disturb functional DNA binding and therefore enable ON and OFF switch design based on simple protein fusion. While many alternative DBDs are available, the number of effective EBDs is limited. By showing that members of one of the largest small-molecule-recognizing protein families are potential candidates for inducible dimerization through their EBDs, we have greatly expanded the toolbox available to build complex logic gates. Furthermore, we were able to identify minimal parts that can be coupled in various combinations, similar to parts in electronic designs, to build a great variety of logic computational networks within a single mammalian cell.

## Methods

### Plasmid design and molecular cloning

All plasmids used in this study were designed using Benchling (www.benchling.com) and are listed in Supplementary Table 1. The sequences of all plasmids were verified at Microsynth AG.

The plasmids were propagated in the bacterial strain XL10-gold K12 *E. coli* (Stratagene). Bacteria were grown at 37 °C in Lurie-Bertani lysogeny broth under shaker-aeration for 8–16 h before plasmid DNA was extracted using a Zippy Plasmid MiniPrep Kit (D4037, Zymo Research) according to the manufacturer’s instructions. PCR reactions were performed using Phusion High-Fidelity DNA polymerase (F530, Thermo Fisher Scientific) according to the manufacturer’s instructions.

For digestion ligation cloning, we digested 300–1,000 ng of plasmid DNA or purified PCR-amplified sequences for 2 h, using 2 units of standard restriction enzyme (New England BioLabs) and calf intestinal alkaline phosphatase (Quick CIP, M0525, New England BioLabs) according to the manufacturer’s instructions. Standard agarose gel electrophoresis was used to purify the digestion products. To extract the DNA fragments, a Zymoclean Gel DNA Recovery Kit (D4002, Zymo Research) was used according to the manufacturer’s instructions. T4 DNA ligase (EL0011, Thermo Fisher Scientific) was then used to ligate the purified DNA fragments to the respective counterparts with a matching overhang in a T4 DNA ligase (B69, Thermo Fisher Scientific) buffered solution for at least 20 min before transformation into competent bacteria. Transformation was achieved by adding 10 µl of the ligation mix to 20–50 µl of competent bacteria. The mixture was then heat-shocked at 42 °C for 45 s and plated onto ampicillin-containing LB-agar. Bacteria were incubated for 16 h on the LB-agar, then a colony was picked and grown as previously described.

### Inducers

All inducers used are listed with the concentration used (unless otherwise stated) in parentheses as follows: Dox (1 µM) (D9891, Sigma-Aldrich), rapamycin (50 nM) (553210, Sigma-Aldrich), abscisic acid (40 µM) (ALX-350-255-M001, Enzo Life Science), GA (40 µM) (G7645, Sigma-Aldrich), VA (250 µM) (H36001, Sigma-Aldrich), d-lactate (25 mM) (71716, Sigma-Aldrich), acetoin (10 mM) (B0753, Sigma-Aldrich), d-idonate (1 mM) (MI08254, CarboSynth), pristinamycin (7.5 µM) (3567278, Bio-Rad), virstatin (50 µM) (358715, ChemCruz), xylose (5 mM) (X3877, Sigma Aldrich).

### Cell culture and transfection

Human embryonic kidney cells (HEK293T, DSMZ: ACC 635) were cultured in Dulbecco’s modified Eagle’s medium (Gibco DMEM, 31966-021, Thermo Fisher Scientific) supplemented with 10% (v/v) fetal bovine serum (FBS, F7524, Sigma-Aldrich) with 1% (vol/vol) streptomycin/penicillin (Gibco Penicillin–Streptomycin, 15070-063, Thermo Fisher Scientific). For all transient transfections, 10,000 cells were seeded 24 h before transfection. The cell density was analyzed using a cell counter (DeNovix CellDrop BF, Labgene Scientific SA). Cells were transfected overnight using 150 ng of DNA per well in a 96-well plate and polyethyleneimine (PEI, 24765-2, Polysciences) in a ratio of 1:6. A PEI stock solution was prepared using 1 mg ml^−1^ PEI in ddH_2_O and stored at −20 °C. The culture medium was exchanged on the following morning for an inducer-containing or inducer-free medium. Cells were then incubated for 24 h before the supernatant was collected for quantification of secreted reporter protein. All transfections are listed in Supplementary Table 2 with the exact transfection mix used per well of a 96-well plate.

### Stable cell line generation

The TetO_7_-SEAP construct was cloned on a Tier-3 vector flanked by Sleeping Beauty transposase recognition sites^46^ and also encoded constitutive expression of the puromycin resistance gene. It was transfected into HEK293T cells capitalizing on the Sleeping Beauty transposon protocol^47^. In short, 25,000 HEK293T cells were seeded into one well of a 6-well plate. Twenty-four hours later, the cells were incubated overnight with a transfection mixture containing pAB482 (800 ng), pTS395 (400 ng) and pDF101 (1,200 ng). The next morning, the medium was replaced with a fresh cell culture medium. Antibiotic selection was started 24 h later by adding 2 µg ml^−1^ puromycin (A1113803; Thermo Fisher Scientific).

### SEAP reporter assay

Secreted placental alkaline phosphatase (SEAP) reporter assay was performed as previously described^48^. Briefly, 20 µl of culture supernatant was mixed with 80 µl of water and then heat-inactivated at 65 °C for 30 min. Next, 2× SEAP assay buffer (20 mM homoarginine, 1 mM MgCl_2_, 21% (vol/vol) diethanolamine, pH 9.8) was mixed with substrate solution (120 mM p-nitrophenyl phosphate in 2× SEAP assay buffer (Acros Organics BVBA)) at a ratio of 4:1. 100 µl of this mixture was then added to 100 µl of diluted heat-inactivated supernatant and measurement was started immediately. Absorbance at 405 nm was measured using a Tecan Infinite M1000 microplate reader over a period of 30 min to determine the time-dependent increase in absorbance.

### Confluency measurement

HEK293T cells were seeded, transfected and treated with virstatin in a standard 6-well cell culture plate as previously described. Images were taken every 30 min with a Nikon Eclipse Ti microscope equipped with a digital camera system (Hamamatsu, ORCA Flash 4) at ×10 magnification in the phase-contrast mode over a period of 60 h. To measure cell confluency, phase-contrast images were filtered with a standard deviation filter (with a (7 × 7) neighborhood) and then segmented with the Triangle thresholding algorithm^49^, as implemented in scikit-image (https://scikit-image.org/docs/stable/api/skimage.filters.html#skimage.filters.threshold_triangle). The relative area covered by cells was calculated as the total number of pixels above the triangle threshold divided by the total image area. Cell area fraction was then plotted over time. For the representation of growth curves, we selected image series with a starting confluency between 15% and 23%.

### RNA sequencing

RNA was isolated using a Quick-RNA MiniPrep Kit (R1055, Zymo Research) from HEK293T cells that had been collected after transfection as previously described and cultured for 24 h in the presence or absence of virstatin. A TruSeq-stranded mRNA Illumina HT kit v2 was used to prepare the isolated RNA for sequencing. The RNA was then sequenced with NextSeq 500 using Illumina RTA v 2.11.3 with 76 cycles. The acquired data were demultiplexed and the Snakemake workflow was used to perform primary analysis. This workflow includes trimmomatic (v 0.35), alignment to the GRCh38 genome with hisat2 (v 2.1.0), samtools (v 1.9) to sort and index the alignment BAM files, and featureCounts from the Subread package (v 2.0.1) to count reads in the gene ranges, using human Ensembl annotation v105. For secondary analysis in R, the count vectors for all samples were combined into one table. The R package PCAtools was used to check the quality and sample consistency with PCA. In secondary (statistical) analysis, the count table was processed with R scripts using edgeR (v3.32). This provides lists of genes ranked for differential expression by *P* value; the Benjamini–Hochberg adjusted *P* value was used to estimate the false discovery rate. Pathway enrichment analysis was performed with GeneGo Metacore. The RNA-seq data are listed in Supplementary Data.

### Statistical analysis

All presented data are representative of three independent experiments. Standard deviation was used to determine variation, which is displayed as error bars within the plots. Statistical evaluation was conducted to examine the significance of differences between two or multiple datasets using the unpaired two-tailed Student’s *t*-test or one-way ANOVA analysis, respectively. Microsoft Excel for Mac version 16.56 was used for data handling and analysis and Graphpad Prism 8 (GraphPad Software) was used for all statistical evaluations and graphical representations.

### Reporting summary

Further information on research design is available in the Nature Portfolio Reporting Summary linked to this article.

## Online content

Any methods, additional references, Nature Portfolio reporting summaries, source data, extended data, supplementary information, acknowledgements, peer review information; details of author contributions and competing interests; and statements of data and code availability are available at 10.1038/s41589-023-01281-x.

## Supplementary information

Supplementary Information Supplementary Tables 1–3.

Reporting Summary

Supplementary Data RNA sequencing data of HEK293T cells transfected with the ToxT system and cultured in the presence (V1-3) or absence (C1-3) of virstatin.

## Data Availability

All relevant data and the exact conditions including plasmid lists (Supplementary Table 2), transfection protocols (Supplementary Table 3) and Source Data files to reproduce these data are available within this paper and its supplementary information. All plasmid maps have been made publicly available on Benchling (https://benchling.com/adrianbertschi/f_/d45hT0BI-combinatorial-protein-dimerization-enables-precise-multi-input-synthetic-computations/). RNA-seq data have been added in an Excel file ‘RNAseq_Data’ (Supplementary Data) to the submitted Publication. All plasmids and materials used within this study are available upon request. Requests for materials should be made to the corresponding author. Source data are provided with this paper.

## Source data

Source Data Fig. 1 Statistical source data.
Source Data Fig. 2 Statistical source data.
Source Data Fig. 3 Statistical source data.
Source Data Fig. 4 Statistical source data.
Source Data Fig. 5 Statistical source data.
Source Data Fig. 6 Statistical source data.
Source Data Extended Data Fig. 1 Statistical source data.
Source Data Extended Data Fig. 2 Statistical source data.
Source Data Extended Data Fig. 3 Statistical source data.
Source Data Extended Data Fig. 4 Statistical source data.
Source Data Extended Data Fig. 5 Statistical source data.
Source Data Extended Data Fig. 6 Statistical source data.
Source Data Extended Data Fig. 7 Statistical source data.
Source Data Extended Data Fig. 8 Statistical source data.
Source Data Extended Data Fig. 9 Statistical source data.
Source Data Extended Data Fig. 10 Statistical source data.
