# Supplementary material for: Combinatorial protein dimerization enables precise multi-input synthetic computations
Source: Nat Chem Biol. 2023 Mar 9;19(6):767–77. doi: 10.1038/s41589-023-01281-x (PMC10229424; doi:10.1038/s41589-023-01281-x)
Supplement: Supplementary file 1 — Supplementary Tables 1–3. [file 41589_2023_1281_MOESM1_ESM.pdf]

nature chemical biology

Article https://doi.org/10.1038/s41589-023-01281-x

Combinatorial protein dimerization enables precise multi-input synthetic computations

In the format provided by the authors and unedited

Table of Contents

Supplementary Table 1: Truth table of multi-input switches **2**

Supplementary Table 2: Plasmids used and constructed in this work **3**

Supplementary Table 3: Plasmids and amount used for transfection per well of a 96-well plate **21**

References **24**

Supplementary Table 1: Truth table of multi-input switches

Input				Output					
A	B	C	D	I	II	III	IV	V	VI
0	0	0	0	0	0	0	0	0	0
0	0	0	1	0	1	0	0	0	0
0	0	1	0	0	1	0	0	0	0
0	0	1	1	0	1	0	1	0	1
0	1	0	0	0	1	0	1	0	0
0	1	0	1	0	1	0	1	1	0
0	1	1	0	0	1	0	1	1	0
0	1	1	1	0	1	0	1	1	1
1	0	0	0	0	1	0	1	0	0
1	0	0	1	0	1	0	1	1	0
1	0	1	0	0	1	0	1	1	0
1	0	1	1	0	1	0	1	1	1
1	1	0	0	0	1	0	1	0	1
1	1	0	1	0	1	1	1	1	1
1	1	1	0	0	1	1	1	1	1
1	1	1	1	1	1	1	1	1	1

Truth table of biological multi-input switches from Figure 6c-h. The table shows the expected outputs in response to an input for all logic gates described in Figure 6. (I: (A AND B) AND (C AND D); II: (A OR B) OR (C OR D); III: (A AND B) AND (C or D); IV: (A OR B) OR (C AND D); V: (A OR B) AND (C OR D); VI: (A AND B) OR (C AND D))

Supplementary Table 2: Plasmids used and constructed in this work

Plasmid Name	Description	Reference
pDF101	Inert filler plasmid bearing a bacterial T7 promoter driving an inactive ribozyme.<br><br>(P_{T7}-SpAL-sTRSVac)	Ausländer et al. 2016[1]
pMSBB11	Constitutive FKBP expression plasmid.<br><br>(P_{hCMV} -FKBP- pA_{bGH})	(Schmollack et al., unpublished)
pMSBB12	Constitutive FRB expression plasmid.<br><br>(P_{hCMV} -FRB- pA_{bGH})	(Schmollack et al., unpublished)
pLS15	Constitutive STAT3 expression plasmid.<br><br>(P_{hCMV} -STAT3- pA_{bGH})	Schukur et al. 2015[2]
pTS566	Expression vector for SEAP under control of four STAT3 operator sites.<br><br>(4xO_{STAT3}-$P_{hCMVmin}$-SEAP-pA)	Strittmatter et al. unpublished
pLeo619	Constitutive aRR120-EpoR-Il6 expression plasmid.<br><br>(P_{SV40}-igk-aRR120-EpoR-Il6-pA_{bGH})	Scheller et al. 2018[3]
pVH246	Constitutive VanR expression vector.	Haellman et al. 2021[4]

	(P_{mPGK1}-VanR- pA_{bGH})	
pVH228	Expression vector for SEAP under control of the VanR operon.<br><br>(O_{VanO2}-$P_{hCMVmin}$-SEAP-pA)	Haellman et al. unpublished
pVH294	Vanillic acid-inducible Igk-NLuc reporter protein expression vector.<br><br>(O_{VanO2}-$P_{CMVmin-1}$-Igk-NLuc-pA).	Haellman et al. 2021[4]
pAT13	Constitutive PIP-ELK1 expression plasmid.<br><br>(P_{hCMV} -PIP-ELK1- pA_{bGH})	Scheller et al. 2018[3]
pWB24	Constitutive L-tryptophan dependent transactivator TRT expression vector.<br><br>(P_{hCMV} -TRT- pA_{bGH})	Bacchus et al. 2012[5]
pMX181	Constitutive VP16-LgnR expression plasmid<br><br>(P_{SV40}-VP16-LgnR-pA_{bGH})	Strittmatter et al. 2021[6]
pMMH1043	Constitutive VanR-TEV_{split}-N expression plasmid.<br><br>(P_{hCMV} -SigP-VanR-Sec-TEV_{split}-N- pA_{bGH})	Mahameed et al., unpublished
pMMH1044	Constitutive VanR-TEV_{split}-C expression plasmid.<br><br>(P_{hCMV} -SigP-VanR-Sec-TEV_{split}-C- pA_{bGH})	Mahameed et al., unpublished
pSLQ2842	Constitutive GID-VPR-IRES-ZEO and GAI-tagBFP-SadCas9 expression plasmid.<br><br>(P_{CAG} -GID-VPR-IRES-Zeo-WPRE; P_{PGK}-GAI-tagBFP-SadCas9 pA_{bGH})	Gao et al. 2016[7]
pTS2365	Constitutive TetR expression plasmid.<br><br>(P_{hCMV}-TetR-pA_{bGH})	Strittmatter et al. unpublished

pVH074	Constitutive PYL1 expression plasmid.<br><br>(P_{hCMV}-PYL1-pA_{bGH})	Haellman et al. unpublished
pVH075	Constitutive ABI expression plasmid.<br><br>(P_{hCMV}-ABI-pA_{bGH})	Haellman et al. unpublished
pTS1017	Expression vector for SEAP under control of the TetR/rTetR operon.<br><br>(O_{TetO7}-$P_{hCMVmin}$-SEAP-pA)	Haellman et al. 2021[4]
pTS395	P_{hCMV}-driven Sleeping Beauty transposase mammalian expression vector.<br><br>(P_{hCMV}-SB100- pAbGH)	Haellman et al. 2021[4]
pTS1015	SEAP expression vector with a minimal version of the CMV promoter.<br><br>($P_{hCMVmin}$-SEAP-pA_{bGH})	Strittmatter et al. unpublished
pTS2341	Stable integration vector via Sleeping Beauty transposase including a PuroR resistance gene<br><br>(ITR-P_{RPBSA}-PuroR-pA_{p9}-ITR)	Strittmatter et al. unpublished
pTS2367	VP16 transactivation domain expression plasmid.<br><br>(P_{hCMV}-VP16-pA_{bGH})	Strittmatter et al. unpublished
pVH15	VPR transactivation domain expression plasmid.<br><br>(P_{hCMV}-VPR-pA_{bGH})	Haellman et al. unpublished
pOM219	Constitutive XylR-VPR expression plasmid.<br><br>(P_{hCMV}-XylR-VPR-pA_{bGH})	Galvan et al. 2022[8]

pAB001	Constitutive AcoR expression plasmid.<br><br>(P_{hCMV}-AcoR-pA_{bGH})	Bertschi et al. unpublished
pAB013	Constitutive AcoR-VPR expression plasmid.<br><br>(P_{hCMV}-AcoR-VPR-pA_{bGH})	Bertschi et al. unpublished
pAB021	Constitutive TrpR expression plasmid.<br><br>TrpR was PCR-amplified from pWB24 with oAB015 (5' CTGAACTAGTGGTGGTTCTGGTATGAAAAATCAAGAGGAGTCTGGCTGGCAGGCTTTTCTG 3') and oAB016 (5' CTGAGGATCCGCTAGCCTACCCACCGTACTCGTCAATTCCAAGG 3'), restricted using *Spe*I *Bam*HI and inserted into *Spe*I *Bam*HI restricted pVH15.<br><br>(P_{hCMV}-TrpR-pA)	This work
pAB022	Expression vector for SEAP under control of the TrpR operon.<br><br>O_{TRT} was obtained through annealing and phosphorylation of oAB017 (5' CGCGTCTCGAGTTGTAATATTATAGCATTACAATTGTAATATTATAGCATTACAAA 3') and oAB018 (5' TCGATTTGTAATGCTATAATATTACAATTGTAATGCTATAATATTACAACTCGAGA 3') and ligation into *Mlu*I *Xho*I restricted pTS1015.<br><br>(O_{TRT}-$P_{hCMVmin}$-SEAP-pA)	This work
pAB040	Expression vector for SEAP under control of the $TetO_2$ operon.<br><br>O_{TetO2} was obtained through annealing and phosphorylation of oAB070 (5' CGCGTAGTAAAGTCTGCATACGTTCTCTATCACTGATAGGGAGTAAACTCTTCATACGTTCTCTATCACTGATAGGGAGTAAAC 3') and oAB071 (5'	This work

	TCGAGTTTACTCCCTATCAGTGATAGAGAACGTATGAAGAGTTTACTCCCTATCAGTGATAGAGAACGTATGCAGACTTTACTA 3') and ligation into *Mlu*I *Xho*I restricted pTS1015.<br><br>(O_{TetO2}-$P_{hCMVmin}$-SEAP-pA)	
pAB080	Constitutive $TrpR_{DBD}$ expression plasmid.<br><br>$TrpR_{DBD}$ was PCR-amplified from pAB21 with oPW009 (5' GGAATTCACCATGACTAGTAAAAATCAAGAGGAG 3') and oPW010 (5' AGTCGGATCCGCTAGCGTAATTGTTTTCGTACGCGCTGATCTC 3'), restricted using *Eco*RI *Nhe*I and inserted into *Eco*RI *Nhe*I restricted pTS2367.<br><br>(P_{hCMV}-$TrpR_{DBD}$-pA_{bGH})	This work
pAB081	Constitutive $TrpR_{DBD}$-VP16 expression plasmid.<br><br>$TrpR_{DBD}$ was restricted using *Eco*RI and *Nhe*I from pAB80 and inserted into *Eco*RI *Spe*I restricted pTS2367.<br><br>(P_{hCMV}-$TrpR_{DBD}$-VP16-pA_{bGH})	This work
pAB082	Constitutive $AcoR_{DBD}$-VP16 expression plasmid.<br><br>$AcoR_{DBD}$ was PCR-amplified from pAB001 with oPW005 (5' CTGAGAATTCACCATGACTAGTGGTGGTTCTGGTGCCGGCGTGCTTACTTTTCG 3') and oPW006 (5' GCTCTAGACACCGGTGCTAGCTTCTG 3'), restricted using *Eco*RI *Nhe*I and inserted into *EcoRI SpeI* restricted pTS2367.<br><br>(P_{hCMV}-$AcoR_{DBD}$-VP16-pA_{bGH})	This work
pAB083	Constitutive $VanR_{DBD}$-VP16 expression plasmid.	This work

	VanR$_{DBD}$ was PCR-amplified from pVH246 with oWP007 (5' CATGGAATTCACCATGACTAGTGGTGGTTCTGGTGACATG 3') and oPW008 (5' CTGAGGATCCGCTAGCGATCTGGTCGCTGGACACGC 3'), restricted using *Eco*RI *Nhe*I and inserted into *Eco*RI *Spe*I restricted pTS2367.<br><br>(P$_{hCMV}$-VanR$_{DBD}$-VP16-pA$_{bGH}$)	
pAB084	Constitutive TetR$_{DBD}$-VP16 expression plasmid.<br><br>TetR$_{DBD}$ was PCR-amplified from pTS2365 with oWP003 (5' CCGGAATTCACCATGACTAGTGGTGG 3') and oPW004 (5' CTGAGGATCCGCTAGCTTCTAAAGGGCAAAAGTGAGTATGGTGC 3'), restricted using *Eco*RI *Nhe*I and inserted into *Eco*RI *Spe*I restricted pTS2367.<br><br>(P$_{hCMV}$-TetR$_{DBD}$-VP16-pA$_{bGH}$)	This work
pAB090	Constitutive VanR-VP16 expression plasmid.<br><br>VanR was restricted using *Eco*RI and *Nhe*I from pVH246 and inserted into *Eco*RI *Spe*I restricted pTS2367.<br><br>(P$_{hCMV}$-VanR-VP16-pA$_{bGH}$)	This work
pAB091	Constitutive VanR$_{[6-]}$-VP16 expression plasmid.<br><br>VanR$_{[6-]}$ was PCR-amplified from pAB090 with oAB060 (5' CTGAACTAGTGGTGGTTCTGGTATAAAGCCGGGCCAGCGCGT 3') and oAB061 (5' TCTAGACACCGGTGGATCCGCTAGC 3'), restricted using *Spe*I *Bam*HI and inserted into *Spe*I *Bam*HI restricted pVH15.<br><br>(P$_{hCMV}$- VanR$_{[6-]}$-VP16 -pA$_{bGH}$)	This work

pAB092	Constitutive VanR$_{[12-]}$-VP16 expression plasmid.<br><br>VanR$_{[12-]}$ was PCR-amplified from pAB090 with oAB062 (5' CTGAACTAGTGGTGGTTCTGGTGTGATGATGGCTCTTCGCAAGATGATCGCC 3') and oAB061 (5' TCTAGAcACCGGTGGATCCGCTAGC 3'), restricted using *Spe*I *Bam*HI and inserted into *Spe*I *Bam*HI restricted pVH15.<br><br>(P$_{hCMV}$- VanR$_{[12-]}$-VP16 -pA$_{bGH}$)	This work
pAB093	Constitutive VanR$_{[17-]}$-VP16 expression plasmid.<br><br>VanR$_{[17-]}$ was PCR-amplified from pAB090 with oAB063 (5' CTGAACTAGTGGTGGTTCTGGTCGCAAGATGATCGCCTCGGGCGAG 3') and oAB061 (5' TCTAGACACCGGTGGATCCGCTAGC 3'), restricted using *Spe*I *Bam*HI and inserted into *Spe*I *Bam*HI restricted pVH15.<br><br>(P$_{hCMV}$- VanR$_{[17-]}$-VP16 -pA$_{bGH}$)	This work
pAB094	Constitutive VanR$_{[22-]}$-VP16 expression plasmid.<br><br>VanR$_{[22-]}$ was PCR-amplified from pAB090 with oAB064 (5' CTGAACTAGTGGTGGTTCTGGTTCGGGCGAGATCAAGAGCGG 3') and oAB061 (5' TCTAGACACCGGTGGATCCGCTAGC 3'), restricted using *Spe*I *Bam*HI and inserted into *Spe*I *Bam*HI restricted pVH15.<br><br>(P$_{hCMV}$- VanR$_{[22-]}$-VP16 -pA$_{bGH}$)	This work
pAB098	Constitutive PIP expression plasmid.<br><br>PIP was PCR-amplified from pAT13 with oPW023 (5' CGAAGCGGAATTCACCATGACTAGTATGAGTCGAGGAGAGGTGCGC 3') and oAB055 (5' CTGAGGATCCGCTAGCGGCCTGTTCGACCATCGCGTC 3'), restricted using *Spe*I *Bam*HI and inserted into *Spe*I *Bam*HI restricted pVH15.	This work

	(P_{hCMV}-PIP-pA_{bGH})	
pAB099	Constitutive Igk-nLuc expression plasmid.<br><br>Igk-nLuc was restricted using *Eco*RI and *Nhe*I from pVH294 and inserted into *Eco*RI *Nhe*I restricted pMX181.<br><br>(P_{SV40}-Igk-nLuc-pA_{bGH})	This work
pAB101	Expression vector for SEAP under control of the AcoR operon.<br><br>(P_{OAcoR}-$P_{hCMVmin}$-SEAP- pA_{bGH})	Bertschi et al. unpublished
pAB300	Constitutive rTetR expression plasmid.<br><br>(P_{hCMV}-rTetR-pA_{bGH})	Bertschi et al. unpublished
pAB301	Constitutive rTetR-VPR expression plasmid.<br><br>(P_{hCMV}-rTetR-VPR-pA_{bGH})	Bertschi et al. unpublished
pAB400	Constitutive D-LldR-TetR expression plasmid.<br><br>D-LldR was obtained from *Pseudomonas fluorescens* (WP_073523526). The nucleotide sequence was human-optimized and synthesized via TWIST with the flanking regions of (5' CATGACTAGTGGTGGTTCTGGT 3') N-terminal and (5' GCTAGCGGATCCTCAG 3') C-terminal, restricted using *Eco*RI and *Nhe*I and inserted into *Eco*RI *Spe*I restricted pTS2365<br><br>(P_{hCMV}-D-LldR-TetR-pA_{bGH})	This work
pAB401	Constitutive D-LldR-VPR expression plasmid.	This work

	D-LldR was obtained from *Pseudomonas fluorescens* (WP_073523526). The nucleotide sequence was human-optimized and synthesized via TWIST with the flanking regions of (5' CATGACTAGTGGTGGTTCTGGT 3') N-terminal and (5' GCTAGCGGATCCTCAG 3') C-terminal, restricted using *Eco*RI and *Nhe*I and inserted into *Eco*RI *Spe*I restricted pVH15<br><br>(P_{hCMV}-D-LldR-VPR-pA_{bGH})	
pAB402	Constitutive $TrpR_{DBD}$-D-LldR expression plasmid.<br><br>D-LldR was obtained from *Pseudomonas fluorescens* (WP_073523526). The nucleotide sequence was human-optimized and synthesized via TWIST with the flanking regions of (5' CATGACTAGTGGTGGTTCTGGT 3') N-terminal and (5' GCTAGCGGATCCTCAG 3') C-terminal, restricted using *SpeI* and *BamHI* and inserted into *NheI BamHI* restricted pAB080<br><br>(P_{hCMV}-$TrpR_{DBD}$-D-LldR-pA_{bGH})	This work
pAB403	Constitutive VanR-TetR expression plasmid.<br><br>VanR was restricted using *Eco*RI and *Nhe*I from pVH246 and inserted into *Eco*RI *Spe*I restricted pTS2365.<br><br>(P_{hCMV}-VanR-TetR-pA_{bGH})	This work
pAB404	Constitutive TetR-VanR expression plasmid.<br><br>VanR was restricted using *Spe*I and *Bam*HI from pVH246 and inserted into *Nhe*I *Bam*HI restricted pTS2365.<br><br>(P_{hCMV}-TetR-VanR-pA_{bGH})	This work
pAB405	Constitutive VanR-VPR expression plasmid.	This work

	VanR was restricted using *Eco*RI and *Nhe*I from pVH246 and inserted into *Eco*RI *Spe*I restricted pVH15.<br><br>(P_{hCMV}-VanR-VPR-pA_{bGH})	
pAB406	Constitutive VPR-VanR expression plasmid.<br><br>VanR was restricted using *Spe*I and *Bam*HI from pVH246 and inserted into *Nhe*I *Bam*HI restricted pVH15.<br><br>(P_{hCMV}-VPR-VanR-pA_{bGH})	This work
pAB407	Constitutive VanR-$TrpR_{DBD}$ expression plasmid.<br><br>VanR was restricted using *Eco*RI and *Nhe*I from pVH246 and inserted into *Eco*RI *Spe*I restricted pAB080.<br><br>(P_{hCMV}-VanR-$TrpR_{DBD}$-pA_{bGH})	This work
pAB408	Constitutive TetR-PIP expression plasmid.<br><br>PIP was restricted using *Spe*I and *Bam*HI from pAB098 and inserted into *Nhe*I *Bam*HI restricted pTS2365.<br><br>(P_{hCMV}-TetR-PIP-pA_{bGH})	This work
pAB409	Constitutive VPR-PIP expression plasmid.<br><br>PIP was restricted using *Spe*I and *Bam*HI from pAB098 and inserted into *Nhe*I *Bam*HI restricted pVH15.<br><br>(P_{hCMV}-VPR-PIP-pA_{bGH})	This work
pAB410	Constitutive $TrpR_{DBD}$-PIP expression plasmid.	This work

	PIP was restricted using *Spe*I and *Bam*HI from pAB098 and inserted into *Nhe*I *Bam*HI restricted pAB080.<br><br>(P_{hCMV}-$TrpR_{DBD}$-PIP-pA_{bGH})	
pAB411	Constitutive AcoR-TetR expression plasmid.<br><br>AcoR was restricted using *EcoRI* and *NheI* from pAB001 and inserted into *Eco*RI *Spe*I restricted pTS2365.<br><br>(P_{hCMV}-AcoR-TetR-pA_{bGH})	This work
pAB413	Constitutive AcoR-$TrpR_{DBD}$ expression plasmid.<br><br>AcoR was restricted using *Eco*RI and *Nhe*I from pAB001 and inserted into *Eco*RI *Spe*I restricted pAB080.<br><br>(P_{hCMV}-AcoR-$TrpR_{DBD}$-pA_{bGH})	This work
pAB414	Constitutive LgnR-TetR expression Vector.<br><br>LgnR was PCR-amplified from pMX181 with oAB056 (5' CTGAGGATCCGCTAGCAGGGTGCAGGCGATAGCCCAGG 3') and oPW028 (5' CGAAGCGGAATTCACCATGACTAGTATGGAGAACGACGCCAATAAGGC 3'), restricted using *Spe*I *Bam*HI and inserted into *Nhe*I *Bam*HI restricted pTS2365.<br><br>(P_{hCMV}-LgnR-TetR-pA_{bGH})	This work
pAB415	Constitutive LgnR-VPR expression Vector.<br><br>LgnR was PCR-amplified from pMX181 with oAB056 (5' CTGAGGATCCGCTAGCAGGGTGCAGGCGATAGCCCAGG	This work

	3') and oPW028 (5' CGAAGCGGAATTCACCATGACTAGTATGGAGAACGACGCCAATAAGGC 3'), restricted using *Spe*I *Bam*HI and inserted into *Nhe*I *Bam*HI restricted pVH15.<br><br>(P_{hCMV}-LgnR-VPR-pA_{bGH})	
pAB416	Constitutive VPR-LgnR expression Vector.<br><br>LgnR was PCR-amplified from pMX181 with oAB056 (5' CTGAGGATCCGCTAGCAGGGTGCAGGCGATAGCCCAGG 3') and oPW028 (5' CGAAGCGGAATTCACCATGACTAGTATGGAGAACGACGCCAATAAGGC 3'), restricted using *Eco*RI *Nhe*I and inserted into *Eco*RI *Spe*I restricted pVH15.<br><br>(P_{hCMV}-VPR-LgnR-pA_{bGH})	This work
pAB417	Constitutive LgnR-$TrpR_{DBD}$ expression Vector.<br><br>LgnR was PCR-amplified from pMX181 with oAB056 (5' CTGAGGATCCGCTAGCAGGGTGCAGGCGATAGCCCAGG 3') and oPW028 (5' CGAAGCGGAATTCACCATGACTAGTATGGAGAACGACGCCAATAAGGC 3'), restricted using *Spe*I *Bam*HI and inserted into *Nhe*I *Bam*HI restricted pAB080.<br><br>(P_{hCMV}-LgnR-$TrpR_{DBD}$-pA_{bGH})	This work
pAB419	Constitutive $TrpR_{DBD}$-FKBP expression plasmid.<br><br>FKBP was restricted using *Spe*I and *Bam*HI from pMSBB11 and inserted into *Nhe*I *Bam*HI restricted pAB080.<br><br>(P_{hCMV}-$TrpR_{DBD}$-FKBP-pA_{bGH})	This work
pAB420	Constitutive FRB-VPR expression plasmid.	This work

	FRB was restricted using *Eco*RI and *Nhe*I from pMSBB12 and inserted into *Eco*RI *Spe*I restricted pVH15.<br><br>(P_{hCMV}-FRB-VPR-pA_{bGH})	
pAB421	Constitutive $TrpR_{DBD}$-ABI expression plasmid.<br><br>ABI was restricted using *Spe*I and *Bam*HI from pVH075 and inserted into *Nhe*I *Bam*HI restricted pAB080.<br><br>(P_{hCMV}-$TrpR_{DBD}$-ABI-pA_{bGH})	This work
pAB422	Constitutive PYL1-VPR expression plasmid.<br><br>PYL1 was restricted using *Eco*RI and *Nhe*I from pVH074 and inserted into *Eco*RI *Spe*I restricted pVH15.<br><br>(P_{hCMV}-PYL1-VPR-pA_{bGH})	This work
pAB423	Constitutive ToxT-TetR expression plasmid.<br><br>ToxT was obtained from the *Vibro cholera* ToxT protein (uniport, MS6_RS03535). The nucleotide sequence was human-optimized and synthesized via TWIST with the flanking regions of (5' CATGACTAGTGGTGGTTCTGGT 3') N-terminal and (5' GCTAGCGGATCCTCAG 3') C-terminal, restricted using *Eco*RI and *Nhe*I and inserted into *Eco*RI *Spe*I restricted pTS2365.<br><br>(P_{hCMV}-ToxT-TetR-pA)	This work
pAB424	Constitutive ToxT-VPR expression plasmid.<br><br>ToxT was obtained from the *Vibro cholera* ToxT protein (uniport, MS6_RS03535). The nucleotide sequence was human-optimized and synthesized via TWIST with the flanking regions of (5'	This work

	CATGACTAGTGGTGGTTCTGGT 3') N-terminal and (5' GCTAGCGGATCCTCAG 3') C-terminal, restricted using *Eco*RI and *Nhe*I and inserted into *Eco*RI *Spe*I restricted pVH15.<br><br>(P_{hCMV}-ToxT-VPR-pA)	
pAB425	Constitutive rTetR-$TrpR_{DBD}$ expression plasmid.<br><br>rTetR was restricted using *Eco*RI and *Nhe*I from pAB300 and inserted into *Eco*RI *Spe*I restricted pAB080.<br><br>(P_{hCMV}-rTetR-$TrpR_{DBD}$-pA_{bGH})	This work
pAB426	Constitutive aRR120-EpoR-IL6-CS_{TEV}-$TrpR_{DBD}$-VP16 expression plasmid.<br><br>aRR120-EpoR-IL6-CS_{TEV} was PCR-amplified from pLeo619 with oAB074 (5' ACTGACGCGTGATCTGCGATCTGCATCTC 3') and oAB075 (5' ACTGACTAGTAGAGCCGCCGGATTGAAAGTACAGGTTCTCAGAGCCGCCCTGAGGCATGT AGCCGCCTTGC 3'), restricted using *Mlu*I *Spe*I and inserted into *Mlu*I *Spe*I restricted pAB081.<br><br>(P_{SV40}-igk-aRR120-EpoR-IL6-CS_{TEV}-$TrpR_{DBD}$-VP16-pA)	This work
pAB427	Constitutive VanR-EpoR-IL6 expression plasmid.<br><br>VanR was PCR-amplified from pVH246 with oAB076_Mlu_for (5' CTGAGGATCCGGTGGTTCTGGTGACATGCCGCGCATAAAGCCG 3') and oAB077 (5' GACTGAATTCACTAGcGGAACCCCCCCCTC 3'), restricted using *Bam*HI *Eco*RI and inserted into *Bam*HI *Eco*RI restricted pLeo619.<br><br>(P_{SV40}-igk-VanR-EpoR-IL6-pA)	This work
pAB428	Expression vector for $TrpR_{DBD}$-D-LldR under control of the TetR/rTetR operon.	This work

	$TrpR_{DBD}$-D-LldR was restricted using *Spe*I and *Bam*HI from pAB402 and inserted into *Spe*I *Bam*HI restricted pVH254.<br><br>(Tet_{O7}-P_{min}-$TrpR_{DBD}$-D-LldR)	
pAB429	Expression vector for D-LldR-VPR under control of the VanR operon.<br><br>D-LldR-VPR was restricted using *Spe*I and *Bam*HI from pAB401 and inserted into *Spe*I *Bam*HI restricted pVH228.<br><br>(Van_{O2}-$P_{hCMVmin}$-D-LldR-VPR)	This work
pAB430	Constitutive FRB-ABI expression plasmid.<br><br>FRB was restricted using *Eco*RI and *Nhe*I from pMMBB12 and inserted into *Eco*RI *Spe*I restricted pVH075.<br><br>(P_{hCMV}-FRB-ABI-pA_{bGH})	This work
pAB431	Constitutive VanR-FKBP expression plasmid.<br><br>VanR was restricted using *Eco*RI and *Nhe*I from pVH246 and inserted into *Eco*RI *Spe*I restricted pMSBB11.<br><br>(P_{hCMV}-VanR-FKBP-pA_{bGH})	This work
pAB433	Constitutive VanR-ABI expression plasmid.<br><br>VanR was restricted using *Eco*RI and *Nhe*I from pVH246 and inserted into *Eco*RI *Spe*I restricted pVH075.	This work

	(P_{hCMV}-VanR-ABI-pA_{bGH})	
pAB434	Constitutive rTetR-ABI expression plasmid.<br><br>ABI was restricted using *Spe*I and *Bam*HI from pVH075 and inserted into *Nhe*I *Bam*HI restricted pAB300.<br><br>(P_{hCMV}-rTetR-ABI-pA_{bGH})	This work
pAB435	Constitutive FKBP-VanR expression plasmid.<br><br>FKBP was restricted using *Eco*RI and *Nhe*I from pMMBB11 and inserted into *Eco*RI *Spe*I restricted pVH246.<br><br>(P_{hCMV}-FKBP-VanR-pA_{bGH})	This work
pAB436	Constitutive TetR-ABI expression plasmid.<br><br>ABI was restricted using *Spe*I and *Bam*HI from pVH075 and inserted into *Nhe*I *Bam*HI restricted pTS2365.<br><br>(P_{hCMV}-TetR-ABI-pA_{bGH})	This work
pAB437	Constitutive PYL1-GAI expression plasmid.<br><br>ABI was PCR-amplified from pSLQ2842 with oAB078 (5' CTGAACTAGTGGTGGTTCTGGTATGAAGAGAGATCATCATCATCATCATCATCAAGATAAGAAGACTATGATGATG 3') and oAB079 (5' CTGAGGATCCGCTAGCATTAAGGTCGGTGAGCATAGAATCAAGCCACG 3'), restricted using *Spe*I *Bam*HI and inserted into *Nhe*I *Bam*HI restricted pVH074.<br><br>(P_{hCMV}-PYL1-GAI-pA_{bGH})	This work

pAB438	Constitutive GID-VPR expression plasmid.<br><br>GID was PCR-amplified from pSLQ2842 with oAB080 (5' CTGAACTAGTGGTGGTTCTGGTATGGCTGCGAGCGATGAAGTTAATCTTATTGAGAGC 3') and oAB081 (5' CTGAGGATCCGCTAGCACATTCCGCGTTTACAAACGCCGAAATCTC 3'), restricted using *Spe*I *Bam*HI and inserted into *Eco*RI *Spe*I restricted pVH15.<br><br>(P_{hCMV}-GID-VPR-pA_{bGH})	This work
pAB480	Constitutive VPR-FRB-$TrpR_{DBD}$-FKBP expression plasmid.<br><br>VPR was restricted using *Eco*RI and *Nhe*I from pVH15 and inserted into *Eco*RI *Spe*I restricted pMSBB12. In a second step, VPR-FRB was then restricted from the resulting plasmid using *Eco*RI and *Nhe*I and inserted into *Eco*RI *Spe*I restricted pAB419.<br><br>(P_{hCMV}-VPR-FRB-$TrpR_{DBD}$-FKBP-pA_{bGH}).	This work
pAB481	Constitutive ABI-$TrpR_{DBD}$-PYL1-VPR expression plasmid.<br><br>PYL1-VPR was restricted using *Spe*I and *Bam*HI from pAB422 and inserted into *Nhe*I *Bam*HI restricted pAB080 resulting in a plasmid constitutively expressing $TrpR_{DBD}$-PYL1-VPR. In a second step, ABI was restricted using *Eco*RI and *Nhe*I from pVH75 and inserted into the *Eco*RI *Spe*I restricted plasmid obtained from step one.<br><br>(P_{hCMV}-ABI-$TrpR_{DBD}$-PYL1-VPR-pA_{bGH}).	This work
pAB482	Stable Sleeping Beauty integration plasmid bearing the reporter vector driving SEAP expression under control of the TetO7 operator and a puromycin resistance gene.	This work

	O_{TetO7}-P_{min}-SEAP was restricted using *Mlu*I and *Nhe*I form pVH254 and was then inserted in the corresponding site of pTS2341 that was previously restricted with *Mlu*I and *Nhe*I.<br><br>(ITR-O_{TetO7}-P_{min}-SEAP-P_{RPBSA}-PuroR-pA_{p9}-ITR)	
pWP004	Constitutive VanR-rTetR expression plasmid.<br><br>VanR was restricted using *Eco*RI and *Nhe*I from pVH246 and inserted into *Eco*RI *Spe*I restricted pAB300.<br><br>(P_{hCMV}-VanR-rTetR-pA_{bGH})	This work
pWP005	Constitutive TetR-FKBP expression plasmid.<br><br>FKBP was restricted using *Spe*I and *Bam*HI from pMSBB11 and inserted into *Nhe*I *Bam*HI restricted pTS2365.<br><br>(P_{hCMV}-TetR-FKBP-pA_{bGH})	This work
pWP006	Constitutive rTetR-FKBP expression plasmid.<br><br>FKBP was restricted using *Spe*I and *Bam*HI from pMSBB11 and inserted into *Nhe*I *Bam*HI restricted pAB300.<br><br>(P_{hCMV}-rTetR-FKBP-pA_{bGH})	This work
pSG170	Constitutive XylR-TetR expression plasmid.<br><br>TetR was PCR-amplified from pTS2365 with oSG191<br>(5’GGTTCTGGTTCCAGATTAGATAAAAGTAAAGTG 3’) and<br>oSG195 (5’ACACCGGTGGATCCGCTAGCGGACCCACTTTCACATTTAA 3’) and cloned into	This work

	pOM219 PCR-amplified with oSG007 (5'CATGGTGAATTCCGCTTCGAAC 3') and oSG190 (5'TCTAATCTGGAACCAGAACCGAGCATCACCTCTGAGTTCA 3') with Gibson assembly.<br><br>(P_{hCMV}-XylR-VPR-pAbGH).	

Supplementary Table 3: Plasmids and amount used for transfection per well of a 96-well plate

Figure 1b	From top to bottom represents from left to right in the figure:<br>30 ng pAB400, 30 ng pAB401, 30 ng pVH254, 60 ng pDF101;<br>30 ng pAB403, 30 ng pAB405, 30 ng pVH254, 60 ng pDF101;<br>30 ng pAB408, 30 ng pAB409, 30 ng pVH254, 60 ng pDF101;<br>30 ng pAB411, 30 ng pAB013, 30 ng pVH254, 60 ng pDF101;<br>30 ng pAB414, 30 ng pAB416, 30 ng pVH254, 60 ng pDF101;
Figure 1c	From top to bottom represents from left to right in the figure:<br>30 ng pAB081, 30 ng pAB022, 90 ng pDF101;<br>30 ng pAB082, 30 ng pAB101, 90 ng pDF101;<br>30 ng pAB083, 30 ng pVH228, 90 ng pDF101;<br>30 ng pAB084, 30 ng pVH254; 90 ng pDF101;
Figure 1d	From top to bottom represents from left to right in the figure:<br>30 ng pAB402, 30 ng pAB401, 30 ng pVH254, 60 ng pDF101;<br>30 ng pAB407, 30 ng pAB405, 30 ng pVH254, 60 ng pDF101;<br>30 ng pAB410, 30 ng pAB409, 30 ng pVH254, 60 ng pDF101;<br>30 ng pAB413, 30 ng pAB013, 30 ng pVH254, 60 ng pDF101;

	30 ng pAB417, 30 ng pAB415, 30 ng pVH254, 60 ng pDF101;<br>30 ng pAB425, 30 ng pAB301, 30 ng pVH254, 60 ng pDF101;
Figure 1e	From top to bottom represents from left to right in the figure:<br>30 ng pAB419, 30 ng pAB420, 30 ng pAB022, 60 ng pDF101;<br>30 ng pAB421, 30 ng pAB422, 30 ng pAB022, 60 ng pDF101;
Figure 2a	From top to bottom represents from left to right in the figure:<br>30 ng pAB423, 30 ng pAB424, 30 ng pVH254, 60 ng pDF101;<br>30 ng pAB423, 30 ng pAB424, 30 ng pVH254, 60 ng pDF101;<br>30 ng pAB423, 30 ng pVH254, 90 ng pDF101;<br>30 ng pAB424, 30 ng pVH254, 90 ng pDF101;
Figure 2b	30 ng pAB423, 30 ng pAB424, 30 ng pVH254, 60 ng pDF101;
Figure 2c	30 ng pAB423, 30 ng pAB424, 30 ng pVH254, 60 ng pDF101;
Figure 2d	From top to bottom represents from left to right in the figure:<br>30 ng pSG170, 30 ng pOM219, 30 ng pVH254, 60 ng pDF101;<br>30 ng pSG170, 30 ng pOM219, 30 ng pVH254, 60 ng pDF101;<br>30 ng pOM219, 30 ng pVH254, 90 ng pDF101;<br>30 ng pSG170, 30 ng pVH254, 90 ng pDF101;
Figure 2e	30 ng pSG170, 30 ng pOM219, 30 ng pVH254, 60 ng pDF101;
Figure 2f	30 ng pSG170, 30 ng pOM219, 30 ng pVH254, 60 ng pDF101;
Figure 3b	From top to bottom represents from left to right in the figure:<br>30 ng pAB403, 30 ng pAB090, 30 ng pVH254, 60 ng pDF101;

	30 ng pAB403, 30 ng pAB091, 30 ng pVH254, 60 ng pDF101;<br>30 ng pAB403, 30 ng pAB092, 30 ng pVH254, 60 ng pDF101;<br>30 ng pAB403, 30 ng pAB093, 30 ng pVH254, 60 ng pDF101;<br>30 ng pAB403, 30 ng pAB094, 30 ng pVH254, 60 ng pDF101;
Figure 3d	From top to bottom represents from left to right in the figure:<br>10ng pMMH1043, 10 ng pMMH1044, 10 ng pAB426, 20 ng pAB022, 100 ng pDF101;<br>10 ng pAB427, 30 ng pLS15, 10 ng pTS566, 100 ng pDF101;
Figure 4b	30 ng pAB403, 30 ng pAB405, 30 ng pVH294, 30 ng pVH254, 30 ng pDF101
Figure 4d	5 ng pAB429, 15 ng pAB428, 15 ng pAB022, 5 ng pAB404, 60ng pAB405, 50 ng of pDF101.
Figure 5b	30 ng pAB403, 30 ng pAB405, 30 ng pVH254, 60ng pDF101
Figure 5c	30 ng pAB403, 30 ng pAB435, 30 ng pAB420, 30 ng pVH254, 30ng pDF101
Figure 5d	30 ng pAB403, 30 ng pAB435, 30 ng pAB430, 30 ng pAB422, 30 ng pVH254
Figure 5e	25 ng pAB403, 25 ng pAB435, 25 ng pAB430, 25 ng pAB437, 25 ng pAB438, 25 ng pVH254
Figure 6b	30 ng pAB403, 30 ng pAB435, 30 ng pAB430, 30 ng pAB422, 30 ng pVH254
Figure 6c	1.5 ng pAB403, 2 ng pAB436, 25 ng pWP005, 1 ng pAB301, 1.5 pAB405, 25 ng pAB420, 2 ng pAB422, 30 ng pVH254, 62 ng pDF101
Figure 6d	5 ng pWP004, 2.5 ng pAB433, 15 ng pAB435, 2.5 ng pAB422, 15 ng pAB420, 15 ng pVH254, 95 ng pDF101
Figure 6e	1 ng pAB301, 1 ng pAB403, 1 ng pAB405, 20 ng pWP005, 20 ng pAB430, 8 ng pAB422, 30 ng pVH254, 69 ng pDF101
Figure 6f	15 ng pAB403, 15 ng pAB435, 1.25 ng pAB433, 15 ng pAB420, 5 ng pAB422, 0.8 ng pWP006, 0.3 ng pAB434, 15 ng pVH254, 82.6 ng pDF101
Figure 6g	2 ng pWP004, 2 ng pAB405, 30 ng pWP005, 30 ng pAB430, 15 ng pAB422, 1 ng pWP006, 30 ng pVH254, 40 ng pDF101

References

1. Ausländer, S., Fuchs, D., Hürlemann, S., Ausländer, D. & Fussenegger, M. Engineering a ribozyme cleavage-induced split fluorescent aptamer complementation assay. *Nucleic Acids Research* **44**, e94-e94 (2016).
2. Schukur, L., Geering, B., Hamri, G.C.-E. & Fussenegger, M. Implantable synthetic cytokine converter cells with AND-gate logic treat experimental psoriasis. *Science Translational Medicine* **7**, 318ra201-318ra201 (2015).
3. Scheller, L., Strittmatter, T., Fuchs, D., Bojar, D. & Fussenegger, M. Generalized extracellular molecule sensor platform for programming cellular behavior. *Nat Chem Biol* **14**, 723-729 (2018).
4. Haellman, V., Saxena, P., Jiang, Y. & Fussenegger, M. Rational design and optimization of synthetic gene switches for controlling cell-fate decisions in pluripotent stem cells. *Metabolic Engineering* **65**, 99-110 (2021).
5. Bacchus, W. et al. Synthetic two-way communication between mammalian cells. *Nature Biotechnology* **30**, 991-996 (2012).
6. Strittmatter, T. et al. Gene switch for l-glucose-induced biopharmaceutical production in mammalian cells. *Biotechnology and Bioengineering* **118**, 2220-2233 (2021).
7. Gao, Y. et al. Complex transcriptional modulation with orthogonal and inducible dCas9 regulators. *Nat Methods* **13**, 1043-1049 (2016).
8. Galvan, S., Madderson, O., Xue, S., Teixeira, A.P. & Fussenegger, M. Regulation of Transgene Expression by the Natural Sweetener Xylose. *Advanced Science*, 2203193 (2022).
