# Supplementary material for: Combinatorial protein dimerization enables precise multi-input synthetic computations
Source: Nat Chem Biol. 2023 Mar 9;19(6):767–77. doi: 10.1038/s41589-023-01281-x (PMC10229424; doi:10.1038/s41589-023-01281-x)
Supplement: Supplementary file 2 — Reporting Summary [file 41589_2023_1281_MOESM2_ESM.pdf]

nature research

Corresponding author(s): Prof. Dr. Martin Fussenegger

Last updated by author(s): Dec 23, 2022

Reporting Summary

Nature Research wishes to improve the reproducibility of the work that we publish. This form provides structure for consistency and transparency in reporting. For further information on Nature Research policies, see our Editorial Policies and the Editorial Policy Checklist.

Statistics

For all statistical analyses, confirm that the following items are present in the figure legend, table legend, main text, or Methods section.

n/a	Confirmed	
☐	☒	The exact sample size (*n*) for each experimental group/condition, given as a discrete number and unit of measurement
☐	☒	A statement on whether measurements were taken from distinct samples or whether the same sample was measured repeatedly
☐	☒	The statistical test(s) used AND whether they are one- or two-sided<br>*Only common tests should be described solely by name; describe more complex techniques in the Methods section.*
☒	☐	A description of all covariates tested
☒	☐	A description of any assumptions or corrections, such as tests of normality and adjustment for multiple comparisons
☐	☒	A full description of the statistical parameters including central tendency (e.g. means) or other basic estimates (e.g. regression coefficient) AND variation (e.g. standard deviation) or associated estimates of uncertainty (e.g. confidence intervals)
☐	☒	For null hypothesis testing, the test statistic (e.g. F, t, r) with confidence intervals, effect sizes, degrees of freedom and P value noted<br>*Give P values as exact values whenever suitable.*
☒	☐	For Bayesian analysis, information on the choice of priors and Markov chain Monte Carlo settings
☒	☐	For hierarchical and complex designs, identification of the appropriate level for tests and full reporting of outcomes
☒	☐	Estimates of effect sizes (e.g. Cohen's *d*, Pearson's *r*), indicating how they were calculated

Our web collection on statistics for biologists contains articles on many of the points above.

Software and code

Policy information about availability of computer code

Data collection	Microsoft Excel for Mac version 16.56, Tecan Infinite M1000 (TECAN AG, Maennedorf, Switzerland) was used to collect Luminescence and absorbance data.
Data analysis	Microsoft Excel for Mac version 16.56, GraphPad Prism 8 for MacOS version 8.4.3, edgeR (v3.32)

For manuscripts utilizing custom algorithms or software that are central to the research but not yet described in published literature, software must be made available to editors and reviewers. We strongly encourage code deposition in a community repository (e.g. GitHub). See the Nature Research guidelines for submitting code & software for further information.

Data

Policy information about availability of data

All manuscripts must include a data availability statement. This statement should provide the following information, where applicable:

- Accession codes, unique identifiers, or web links for publicly available datasets
- A list of figures that have associated raw data
- A description of any restrictions on data availability

All relevant data and the exact conditions including plasmid lists (Supplementary Table 2), transfection protocols (Supplementary Table 3) and Source Data files to reproduce these data are available within this paper and its supplementary information. All plasmid maps have been made publicly available on Benchling (https://benchling.com/adrianbertschi/f_/d45hT0BI-combinatorial-protein-dimerization-enables-precise-multi-input-synthetic-computations/). RNAseq data have been added in an Excel file “RNAseq_Data” to the submitted Publication. All plasmids and materials used within this study are available upon request. Requests for materials should be made to the corresponding author.

Field-specific reporting

Please select the one below that is the best fit for your research. If you are not sure, read the appropriate sections before making your selection.

☒ Life sciences ☐ Behavioural & social sciences ☐ Ecological, evolutionary & environmental sciences

For a reference copy of the document with all sections, see nature.com/documents/nr-reporting-summary-flat.pdf

Life sciences study design

All studies must disclose on these points even when the disclosure is negative.

Sample size	There was no statistical methods used to predetermine sample size. We predicted sample size of n=3 biological replicates to be sufficient to estimate the variation and therefore the difference of means. The sample size of n=3 biological replicates is consistent with previously published studies in our field (Scheller L., Nat. Chem. Biol., 2018; Müller M., Nat. Chem. Biol., 2017).
Data exclusions	No data was excluded. All data points obtained during the experiments are shown.
Replication	Each experiment was successfully replicated at least 3 times in independent experiments.
Randomization	All cells within an experiment were treated under the same conditions. There were no covariates observed due to allocations of cells to a sample group. Before starting the experiments, cells were throughly mixed and cell mixture was seeded homogeneously among all groups of the experiment. Wherever possible, the same transfection mix was pipetted to all sample wells of one experiment.
Blinding	The study was not performed blinded as the investigator that performed the manual cell handling was the same investigator that was processing the data and analyzing the results.

Reporting for specific materials, systems and methods

We require information from authors about some types of materials, experimental systems and methods used in many studies. Here, indicate whether each material, system or method listed is relevant to your study. If you are not sure if a list item applies to your research, read the appropriate section before selecting a response.

Materials & experimental systems

n/a	Involved in the study	
☒	☐	Antibodies
☐	☒	Eukaryotic cell lines
☒	☐	Palaeontology and archaeology
☒	☐	Animals and other organisms
☒	☐	Human research participants
☒	☐	Clinical data
☒	☐	Dual use research of concern

Methods

n/a	Involved in the study	
☒	☐	ChIP-seq
☒	☐	Flow cytometry
☒	☐	MRI-based neuroimaging

Eukaryotic cell lines

Policy information about cell lines

Cell line source(s)	Human embryonic kidney cells (HEK293T, DSMZ: ACC 635)
Authentication	HEK293T cells were authenticated by DSMZ and regularly controlled by light microscopy.
Mycoplasma contamination	HEK293T cells were recently obtained mycoplasma-free from the DSMZ cell bank and were not further tested for mycoplasma contamination.
Commonly misidentified lines (See ICLAC register)	Cell lines used are not listed in the ICLAC register as commonly misidentified lines.

April 2020
